# Therapeutic Effects of *Zanthoxyli Pericarpium* on Intestinal Inflammation and Network Pharmacological Mechanism Analysis in a Dextran Sodium Sulfate-Induced Colitis Mouse Model

**DOI:** 10.3390/nu16203521

**Published:** 2024-10-17

**Authors:** Woo-Gyun Choi, Seok-Jae Ko, Daehwa Jung, Sang Chan Kim, Na-Ri Choi, Jae-Woo Park, Byung Joo Kim

**Affiliations:** 1 Department of Longevity and Biofunctional Medicine, Pusan National University School of Korean Medicine, Yangsan 50612, Republic of Korea; ak0510@hanmail.net (W.-G.C.); nariring@gmail.com (N.-R.C.); 2 Department of Clinical Korean Medicine, Graduate School, Kyung Hee University, Seoul 02447, Republic of Korea; kokokoko119@daum.net; 3 Department of Gastroenterology, College of Korean Medicine, Kyung Hee University, Seoul 02447, Republic of Korea; 4 Department of Pharmaceutical Engineering, Daegu Hanny University, Gyeongsan 38610, Republic of Korea; jdh8024@daum.net; 5 College of Oriental Medicine, Daegu Haany University, Gyeongsan 38610, Republic of Korea; sckim@dhu.ac.kr; 6 Department of Korean Medical Science, Pusan National University School of Korean Medicine, Yangsan 50612, Republic of Korea

**Keywords:** inflammatory bowel disease, *Zanthoxyli Pericarpium*, traditional Chinese medicine, network pharmacological, colitis, dextran sodium sulfate

## Abstract

(1) Background: IBD (inflammatory bowel disease) is characterized by chronic intestinal inflammation leading to persistent symptoms and a lack of effective treatments. ZP (*Zanthoxyli Pericarpium*) has been used in traditional Chinese medicine for its anti-inflammatory and antioxidant properties for the management of intestinal disorders. (2) Methods: This study aimed to investigate the components of ZP, their specific targets, and associated diseases using the TCMSP (Traditional Chinese Medicine Systems Pharmacology) analysis platform, TCMBank database, and ETCM2.0 (Encyclopedia of Traditional Chinese Medicine 2.0) database. Additionally, we explored the protective effects of ZP on the colon and the underlying molecular mechanisms in the treatment of IBD. (3) Results: We identified 59 compounds in ZP that target 38 genes related to IBD, including PTGS2, PPARG, and GPBAR1. In a mice model of DSS (dextran sodium sulfate)-induced colitis, ZP significantly reduced colonic epithelial damage and oxidative stress markers, such as iNOS and nitrotyrosine, demonstrating its antioxidant properties. (4) Conclusions: These findings suggest that ZP has protective effects against DSS-induced colonic damage owing to its anti-inflammatory and antioxidant properties, making it a potential candidate for IBD treatment. However, further research and clinical trials are required to confirm its therapeutic potential and safety in humans.

## 1. Introduction

IBD (inflammatory bowel disease), which includes UC (ulcerative colitis) and CD (Crohn’s disease), arises from a combination of genetic predisposition, environmental factors, immune dysregulation, and bacterial dysbiosis. These two distinct disorders, CD and UC, involve a complex interplay between these factors [1,2]. The prevalence of IBD is increasing globally, with notable increases observed in Africa and Asia [3,4]. The development of IBD is thought to result from a combination of factors including genetic predisposition, environmental influences, alterations in the gut microbiota, and disturbances in mucosal immune function. NF-κB/IκB signaling is central to the regulation of cytokine release in patients with UC and is involved in the inflammatory and immune responses in the gut with UC [5]. Existing treatment strategies for IBD primarily revolve around the use of anti-inflammatory medications, such as corticosteroids and formulations based on 5-aminosalicylic acid, azathioprine, and TNF-α inhibitors, including biological agents [6,7]. However, the side effects of these medications, including nausea, diarrhea, and abdominal pain, are not tolerated by some individuals. A significant concern is that a large proportion of patients, despite adopting an aggressive step-down approach, struggle to attain lasting remission without relying on prolonged steroid usage and face the potential need for surgery. Therefore, it is imperative to develop innovative therapeutic approaches for the treatment of IBD.

ZP (*Zanthoxyli Pericarpium*) is derived from the dried pericarp of mature fruits of *Zanthoxylum schinifolium* Siebold and Zucc. (Rutaceae) or *Zanthoxylum bungeanum* Maxim., which are found in Japan, China, and Korea. In traditional Korean medicine, the ZPE (Extract of *Zanthoxyli Pericarpium*) has been utilized to alleviate symptoms such as stomach and spleen discomfort, cold sensations, indigestion, diarrhea, gastritis, and toothache [8].

**Figure 1:**
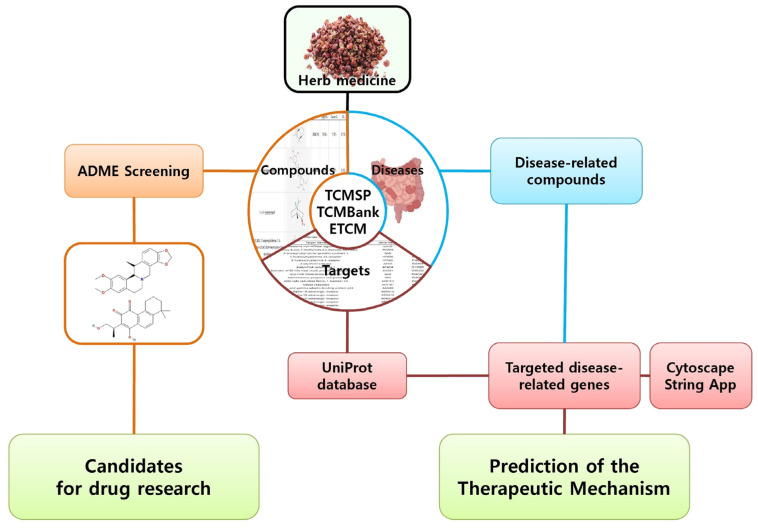
Schematic of the study.

To examine the results and therapeutic mechanisms of ZP in IBD, we utilized a network-based systems pharmacological approach that integrates bioinformatics and computational systems biology. Network pharmacology provides a systems-level analysis of the effects of multi-component drugs, such as herbal medications, on the human body, facilitating the discovery of therapeutic targets for the active pharmaceutical ingredients, enhancing drug efficacy, and minimizing adverse effects [9]. Our initial step involved using the TCMSP (Traditional Chinese Medicine Systems Pharmacology) analysis platform and database, TCMBank database, and ETCM2.0 (Encyclopedia of Traditional Chinese Medicine 2.0) database, which are among several databases and servers that support systems pharmacology. These databases include valuable pharmacokinetic data on ADME (absorption, distribution, metabolism, and excretion) and serve as valuable resources for those seeking to develop new drugs or enhance the effectiveness of herbal remedies. Additionally, we assessed the ZP-compound-target-IBD relationship using other databases and software and predicted the major IBD-related medicinal targets of ZP. An overview of the research protocol is shown in Figure 1.

## 2. Materials and Methods

### 2.1. Identification of Compounds in ZP

We used an analysis platform and database of TCM sites (TCMSP, TCMBank, and ETCM2.0) to identify the potential active compounds of ZP. The TCMSP (https://tcmsp-e.com, accessed on 7 February 2024) provides extensive information on the connections between molecular targets and diseases, drugs and ingredients and pharmacokinetics and molecular structures [10]. TCMBank (https://tcmbank.cn/, accessed on 7 February 2024) offers a comprehensive collection of compounds found in traditional Chinese medicine, along with their pharmacological effects and mechanisms of action [11]. ETCM2.0 (http://www.tcmip.cn/ETCM2/front/#/, accessed on 7 February 2024) provides an integrated platform for exploring traditional Chinese medicine ingredients, targets, and diseases, offering insights into their pharmacokinetics and molecular interactions [12]. We entered ‘*Zanthoxyli Pericarpium*’ into the search bar and searched for the herb name.

### 2.2. Target Analysis

The majority of the target information for the compounds was sourced from TCM sites (TCMSP, TCMBank, and ETCM), and the remaining information was obtained from the Similarity Ensemble Approach server (http://sea.bkslab.org/, accessed on 7 February 2024) [13,14]. To establish a connection between the target proteins and their official gene names, we used the UniProt database (http://www.uniprot.org/, accessed on 7 February 2024) [15].

### 2.3. Network Analysis

We constructed a compound-target network using Cytoscape 3.9.1 (https://cytoscape.org/, accessed on 7 February 2024) [16]. IBD-related genes were identified using the Cytoscape StringApp (Seattle, WA, USA), a tool that integrates weekly updated evidence for gene associations [17].

### 2.4. Active Compound Screening

In our screening of the physiologically active compounds present in ZP, we applied specific criteria based on ADME parameters, including molecular weight, OB (oral bioavailability), DL (drug-likeness), and Caco-2 (Caco-2 permeability). The following criteria were applied: OB ≥ 30%, DL ≥ 0.10, and Caco-2 ≥ −0.4. Compounds meeting these criteria were classified as active compounds. These criteria are based on previous research [18].

### 2.5. Docking Analysis

Docking analysis with AutoDock Vina (v1.2.5) was employed to evaluate the binding affinities of the active compounds to their respective target proteins. The target protein structures (PDB files) were sourced from the PDB (Protein Data Bank, https://www.rcsb.org/, accessed on 7 February 2024), while the structures of the 15 active compounds were retrieved from the PubChem database (https://pubchem.ncbi.nlm.nih.gov/, accessed on 7 February 2024). The retrieved structures for docking analysis were visualized and modified using PyMOL (PyMOL Molecular Graphics System, version 2.5.5; Schrödinger, New York, NY, USA) and Auto-DockTools (version 1.5.7; Molecular Graphics Laboratory, Greensboro, NC, USA).

### 2.6. Preparation of the ZPE

The preparation process of ZPE was the same as that described in a previous study [19]. The ZPE was maintained at the College of Oriental Medicine, Daegu Haany University (voucher specimen number: M230914-1). The standard preparations of this experiment were from xanthoxylin (ChemFaces, Wuhan, China), auraptene (Sigma-Aldrich, St. Louis, MO, USA), and bergapten (Sigma-Aldrich, St. Louis, MO, USA). The bergapten was analyzed at 280 nm in photodiode array detector analysis wavelength. Auraptene and Xanthoxylin were analyzed at 380 nm. The mobile phase consisted of a blend of acetonitrile and water with 0.1% formic acid. The analytical conditions were as described: a 2 μL sample injection and a flow rate of 0.4 mL/min (Supplementary Material Table S1). The results of the analysis were qualitatively checked for retention time and then quantified using the peak area method (Supplementary Material Figure S1 and Supplementary Material Table S2).

### 2.7. Animal Treatment

Male C57BL/6N mice, ranging in age from 6 to 8 weeks, were housed in a controlled lighting environment with a 12-h light/dark cycle. In this experiment, male mice were used to prevent hormonal fluctuations in females from affecting outcomes and causing variability. All the animals had free access to water and food. Acute colitis was induced by administering 3% *w*/*v* DSS (dextran sodium sulfate; Sigma-Aldrich, St. Louis, MO, USA) solution in drinking water for 7 days, followed by a switch to regular drinking water for the subsequent 7 days (Figure 1). This model was determined during preliminary experiments based on the reference [20]. The mice were randomly divided into three groups as follows: (a) the DSS group, which received standard food and access to regular drinking water; (b) the DSS group, which received standard food along with drinking water containing 3% DSS; and (c) the DSS + ZP group, which received ZP (2 g/kg) suspended in 0.5% carboxymethyl cellulose and orally administered with drinking water containing 3% DSS. Each group consisted of *n* = 5–10 mice.

### 2.8. Assessment of Colitis

To assess the severity of colitis induced by DSS, colon tissue specimens were obtained from the cecum junction to the anal margin of euthanized mice. These specimens were rinsed with phosphate-buffered saline to eliminate fecal remnants and subsequently embedded in filter paper. The length of each colon specimen was recorded. The DAI (Disease Activity Index) was computed according to the parameters specified in Supplementary Table S3.

### 2.9. Histological Analysis

To detect subtle alterations in the intestinal and liver tissues, colonic and small intestinal resections were performed on all mice. Excised tissues were fixed in 4% neutral formalin, dehydrated, and embedded in paraffin. Sections of the liver, ileum, and colon, which were embedded in paraffin, were cut at a thickness of 4 μm and forwarded to the Pathology Laboratory at the School of Medicine, Pusan National University, for H&E staining. Damage observed in each liver, small intestine, and colon was evaluated using a scoring system from 0 to 4, based on histological slides stained with H&E (hematoxylin and eosin) [21].

### 2.10. Immunoblot Analysis

For immunoprecipitation analysis, the liver, small intestine, and colon tissue sections from each mouse were homogenized in RIPA buffer. The same amount of protein, derived from pooled samples of multiple mice within each experimental group, was separated using 10% SDS-PAGE and subsequently transferred to nitrocellulose membranes. The nitrocellulose membranes were incubated with specific primary antibodies against Claudin-4 (1:1000; Santa Cruz Biotechnology, Dallas, TX, USA), cyclin-dependent kinase 2 (cdk2; 1:1000; Santa Cruz Biotechnology), cyclin-dependent kinase 4 (cdk4; 1:1000; Santa Cruz Biotechnology), Cyclin D-1 (1:1000; Santa Cruz Biotechnology), COX-2 (1:1000; Santa Cruz Biotechnology), iNOS (1:200; Abcam, Cambridge, UK), p-IκB (1:1000; Santa Cruz Biotechnology), p-NFκB (1:1000; Santa Cruz Biotechnology), 3-NT (1:5000; Abcam), α-tubulin (1:1000; Santa Cruz Biotechnology), β-actin (1:1000; Santa Cruz Biotechnology), or GAPDH (1:1000; Santa Cruz Biotechnology) as specified to detect specific antigen targets. After three 10 min interval washes with TBS-T buffer, the nitrocellulose membranes were incubated with secondary antibodies (anti-mouse or anti-rabbit IgG, Santa Cruz Biotechnology) conjugated to HRP (horseradish peroxidase) at a 1:5000 dilution. Immunoblots were performed using horseradish peroxidase-conjugated secondary antibodies in conjunction with an enhanced chemiluminescence substrate (Thermo Fisher, Waltham, MA, USA). The intensity of the immunoreactive bands was quantified through densitometry analysis using ImageJ software (v1.53; National Institutes of Health).

### 2.11. Statistical Analysis and Other Methods

The data were subjected to statistical analysis using SPSS software (version 27.0; SPSS Inc., Chicago, IL, USA). A *p*-value of less than 0.05 was considered statistically significant for the purpose of evaluating differences in means. Different letters assigned to actual values indicate significant differences among the various treatments, as determined using one-way analysis of variance, with a significance level of *p* < 0.05. Furthermore, Tukey’s HSD (Honestly Significant Difference) test was utilized to evaluate significant intergroup differences. All other methodologies used in this study align with those reported in the recent literature, though they are not elaborated upon here [22,23,24].

## 3. Results

### 3.1. Investigation of the Correlation Between Compounds and Targets Yielded 526 Sources of Target Information

**Figure 2:**
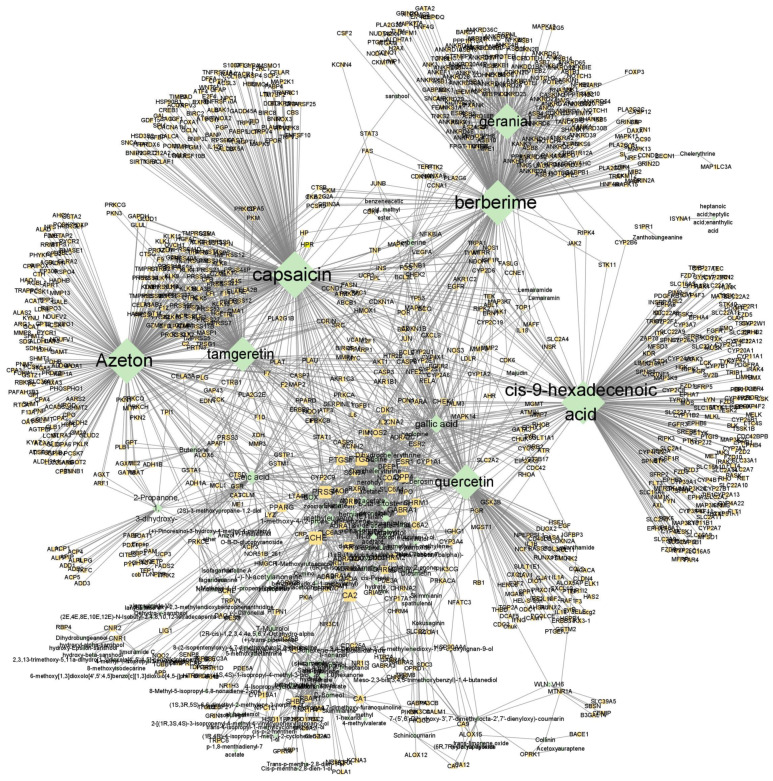
Compound–target network of ZP. In the network, green diamond nodes represent compounds and yellow round nodes represent targets. The size of the nodes reflects the number of connected edges.

A total of 260 potentially active compounds were extracted from ZP based on the data obtained from the TCM site databases (TCMSP, TCMBank, ETCM2.0) (Supplementary Material Table S4). Of these, 131 active compounds had available information on 1121 targets (Supplementary Material Table S5), resulting in 2604 total compound–target interactions (Figure 2). In descending order, as depicted in Figure 2, capsaicin exhibited the highest number of connections to targets (300 genes), followed by berberime (278 genes), Acetone (276 genes), cis-9-hexadecenoic acid (253 genes), geranial (158 genes), quercetin (153 genes), tamgeretin (145 genes), gallic acid (67 genes), oleic acid (48 genes), eucalyptol (39 genes), beta-sitosterol (38 genes), methyleugenol (29 genes), 2-propanone, 1, 3-dihydroxy- (28 genes), (−)-*N*-acetylanonaine (24 genes), suberosin (24 genes), nerol acetate (23 genes), 1-methoxy-4-(2-propenyl)-benzene (23 genes), alpha-terpinol (21 genes), herniarin (21 genes), Dihydrochelerythrine (21 genes), and linalyl anthranilate (20 genes).

### 3.2. Seventeen Active Compounds Met the Criteria for Absorption, Distribution, Metabolism, and Excretion Parameters

To screen for active compounds, specific criteria for ADME parameters were applied, and 131 compounds that satisfied these criteria were defined as active compounds (Table 1).

### 3.3. 37 Targeting Genes of ZP Were Related to IBD

**Figure 3:**
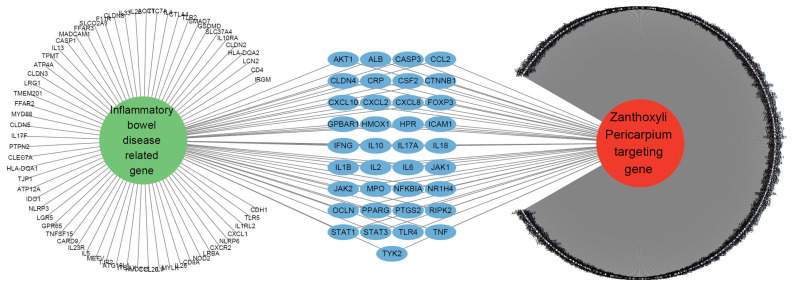
Network of IBD-related genes and ZP target genes.

To explore the connection between ZP and IBD, we utilized Cytoscape StringApp to investigate the gene information associated with IBD. By applying a maximum protein count of 100 and a confidence (score) cutoff of 0.40, we identified 100 IBD-related genes (Supplementary Material Table S6). Based on these findings, we constructed a network depicting the relationships between IBD-related genes and ZP target genes (Figure 3). We identified 37 genes by comparing these two gene sets. Among the IBD-related genes targeted by ZP were AKT1, ALB, CASP3, CCL2, CLDN4, CRP, CSF2, CTNNB1, CXCL10, CXCL2, CXCL8, FOXP3, GPBAR1, HMOX1, HPR, ICAM1, IFNG, IL10, IL17A, IL18, IL1B, IL2, IL6, JAK1, JAK2, MPO, NFKBIA, NR1H4, OCLN, PPARG, PTGS2, RIPK2, STAT1, STAT3, TLR4, TNF, and TYK2.

### 3.4. Network of IBD-Associated Genes and Compounds for Identifying Target Molecules of Interest

**Figure 4:**
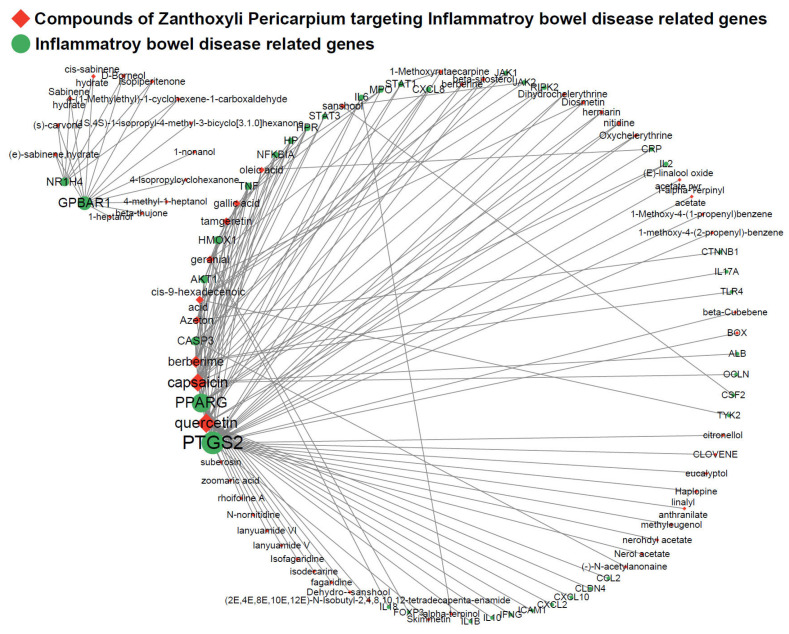
Network of compounds of ZP- and IBD-related genes. In the network, red diamond nodes represent compounds and green round nodes represent targets. The size of the nodes reflects the number of connected edges.

The network diagram presented in Figure 4 shows the connections between the ZP compounds and target genes linked to IBD. Quercetin and PTGS2 exhibited the most robust associations with IBD. In summary, quercetin (21 genes), capsaicin (18 genes), berberime (11 genes), tamgeretin (6 genes), geranial (6 genes), cis-9-hexadecenoic acid (6 genes), Acetone (6 genes), gallic acid (5 genes), oleic acid (4 genes), sanshool (3 genes), berberine (2 genes), beta-sitosterol (2 genes), oxychelerythrine (2 genes), cis-sabinene hydrate (2 genes), Diosmetin (2 genes), herniarin (2 genes), nitidine (2 genes), (s)-carvone (2 genes), 1-methoxyrutaecarpine (2 genes), D-Borneol (2 genes), dihydrochelerythrine (2 genes), isopiperitenone (2 genes), sabinene hydrate (2 genes), 4-(1-methylethyl)-1-cyclohexene-1-carboxaldehyde (2 genes), and (e)-sabinene,hydrate (2 genes) were active compounds targeting IBD-associated genes, suggesting their potential as therapeutic candidates.

### 3.5. Through Docking Analysis, We Identified 15 Active Compounds with Strong Binding Affinity for PTGS2

**Figure 5:**
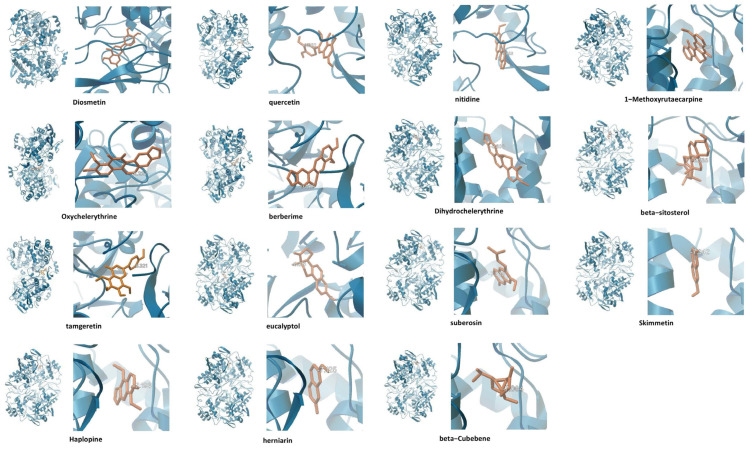
Binding interactions of active compounds with PTGS2 residues. The blue ribbon structure represents the PTGS2 protein, while the orange structures represent the active compounds. The numbers indicate the binding affinity in kcal/mol.

By selecting PTGS2, with the highest binding affinity, as the representative protein, we conducted a docking analysis of 32 active compounds related to PTGS2 to predict their binding affinities. As shown in Table 2, 15 active compounds demonstrated strong binding affinities for PTGS2, with binding energies of less than −7 kcal/mol. Figure 5 illustrates the binding modes of these interactions.

### 3.6. ZPE Significantly Ameliorated the Severity of DSS-Induced Colitis in Mice

**Figure 6:**
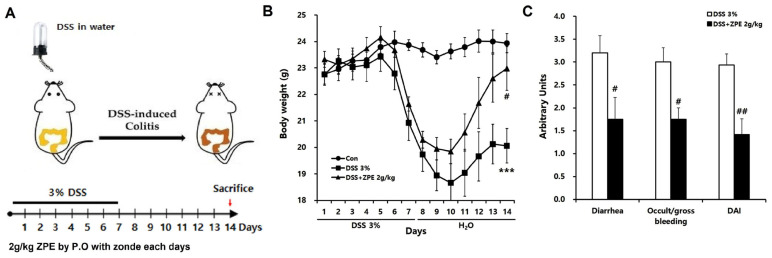
ZPE attenuated the severity of DSS-induced IBD in mice. (**A**) Infographics of the DSS-induced colitis model and the DSS administration cycles; (**B**) body weight of colitis-induced mice administered 3% DSS in drinking water for 7 days and regular drinking water for 7 days was measured daily, and the daily change in mean body weight of each group is shown; (**C**) total DAI score assessed at the end of the experiment in DSS-induced colitis mice with or without ZPE is shown. *** *p* < 0.005 (versus the control group); # *p* < 0.05 and ## *p* < 0.01 (versus the DSS group).

To evaluate the impact of ZPE on intestinal injury, C57BL/6N mice were administered 3% DSS in their drinking water for a seven-day period, followed by a seven-day interval with regular drinking water. Mice exposed to DSS displayed characteristic symptoms of IBD, such as noticeable weight loss and the presence of blood in their stools, as evidenced by their DAI scores. However, the co-administration of ZPE and DSS reduced the DAI scores, occult/gross bleeding, and diarrhea induced by DSS (Figure 6).

### 3.7. Effect of ZPE on DSS-Induced Liver Injury

**Figure 7:**
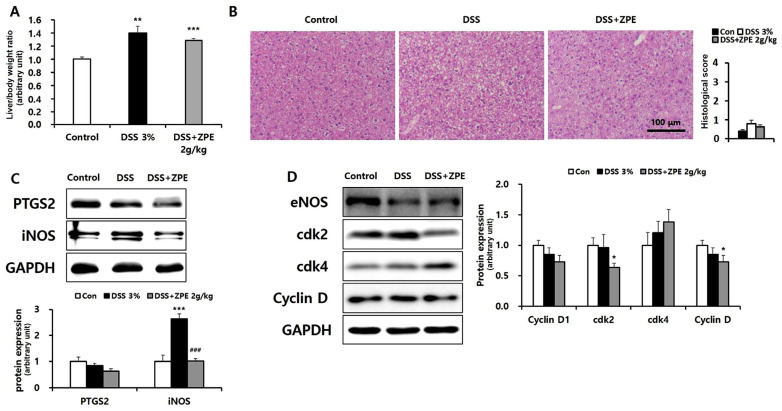
Effect of ZPE on DSS-induced liver injury. (**A**) Liver/body weight ratios are compared; (**B**) micrographs of liver sections with H&E staining and histological score; (**C**,**D**) levels of each target protein in the indicated groups of liver samples are shown. GAPDH is presented for densitometric analysis of the relative abundance of each protein. * *p* < 0.05, ** *p* < 0.01, and *** *p* < 0.005 (versus the control group); ### *p* < 0.005 (versus the DSS group).

Recent studies have indicated that liver damage is commonly seen in patients with IBD. Consequently, this study sought to evaluate the extent of liver injury in DSS-treated mice and investigate the potential protective effects of ZPE administration. Body weight measurements and H&E-stained histological analysis revealed an increase in liver weight in DSS-exposed mice; however, no signs of inflammation according to histopathological analysis were observed, and a non-significant decrease in liver/body weight ratio persisted with ZPE treatment (Figure 7A,B). The expression of the oxidative stress marker PTGS2 showed no significant difference with DSS treatment, and ZPE treatment had a minimal impact. However, in the case of iNOS, an increase in DSS and a decrease in ZPE treatment were observed (Figure 7C). Additionally, the levels of the liver regeneration markers cdk2, cdk4, eNOS, and Cyclin D showed no significant changes after DSS exposure, and there were no significant changes in the levels of these proteins after ZPE treatment (Figure 7D). The findings suggest that liver injury was not pronounced in the DSS-induced colitis model, and the effects of ZPE in this context were not particularly prominent.

### 3.8. Effects of ZPE on Decreased Markers of Oxidative Stress and Inflammatory in DSS-Induced Colitis Mouse Small Intestine

**Figure 8:**
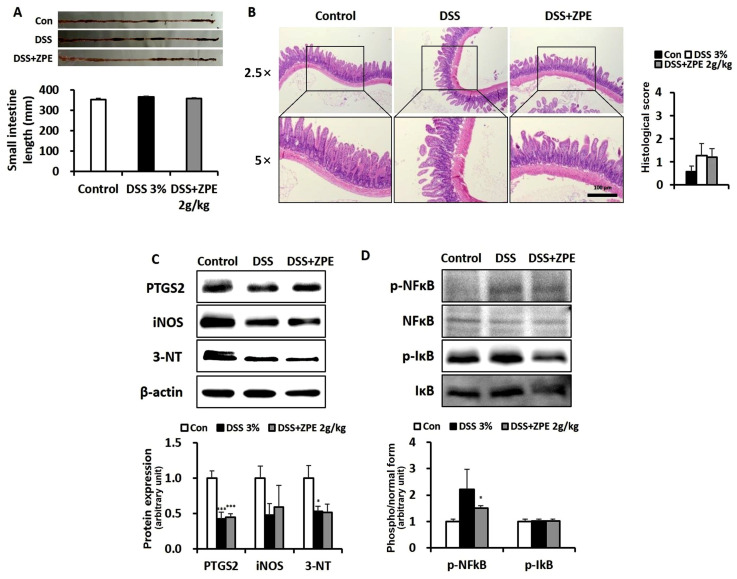
Effects of ZPE on ROS and inflammation in the intestine. (**A**) Comparing the length of the small intestine in each group; (**B**) micrographs of small intestine sections with H&E staining and histological score; (**C**,**D**) levels of each target protein in the indicated groups of small intestine samples. β-actin is presented for densitometric analysis of the relative abundance of each protein. * *p* < 0.05 and *** *p* < 0.005 (versus the control groups).

Several studies have assessed the impact of ZPE on intestinal damage in DSS-induced colitis. These evaluations encompassed measurements of small intestine length and western blot analysis targeting various proteins associated with oxidative stress markers and inflammatory markers. These analyses were performed on different groups of mice. Our findings indicated that DSS and ZPE treatments did not have a significant impact on the length of the small intestine (Figure 8A). Histological analysis of the intestinal epithelium revealed the absence of inflammatory ulcers or microvilli in mice treated with DSS. Furthermore, no noticeable differences were observed after ZPE treatment, as depicted in Figure 8B. The data indicated no elevation in oxidative stress marker proteins in the small intestines of mice exposed to DSS, including PTGS2 and nitrosylated proteins, as detected using the anti-3-NT antibody. Furthermore, no differences were observed with ZPE treatment (Figure 8C). Similarly, there was no observed increase in phosphorylated IκB and NF-κB (nuclear factor-κB) proteins in the small intestines of DSS-exposed mice, and ZPE treatment did not induce any changes (Figure 8D).

### 3.9. Decreased Markers of Oxidative Stress and Inflammation in the ZPE-Treated Colon of DSS-Induced Colitis Mice

**Figure 9:**
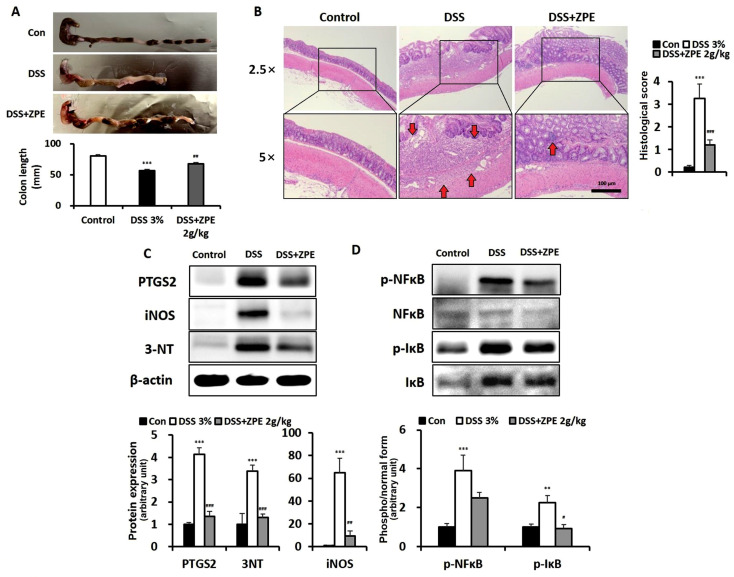
Effects of ZPE on ROS and inflammation in the colon. (**A**) Comparing the length of the colon in each group; (**B**) micrographs of colon sections with H&E staining and histological score (red arrow: inflammatory infiltrate); (**C**,**D**) levels of each target protein in the indicated groups of the colon samples. β-actin is presented for densitometric analysis of the relative abundance of each protein. ** *p* < 0.01 and *** *p* < 0.005 (versus the control group); # *p* < 0.05, ## *p* < 0.01, and ### *p* < 0.005 (versus the DSS group).

To evaluate the therapeutic efficacy of ZPE in a DSS-induced colitis model, colon length was measured. DSS treatment resulted in decreased colon length, whereas ZPE treatment restored it (Figure 9A). Histological analysis of the colon from DSS-induced colitis mice showed ulceration, crypt loss, and inflammation. Treatment with ZPE mitigated the DSS-induced damage and inflammation (Figure 9B). Increased inflammation and markers associated with oxidative stress, such as PTGS2 and iNOS, were present in the colons of mice with DSS-induced colitis. Indeed, the levels of PTGS2, iNOS, and nitrosylated proteins (detected using an anti-3-NT antibody) were increased in the colons of mice with DSS-induced colitis compared to those in the control group. However, ZPE treatment markedly decreased the levels of inflammatory and oxidative stress/nitric oxide marker proteins, such as PTGS2, iNOS, and nitrosylated proteins (Figure 9C). To ascertain the effect of ZPE on the activation of the NF-κB pathway in the colon of mice with DSS-induced colitis, the levels of phosphorylated and unphosphorylated NF-κB and IκB proteins were quantified. The results revealed a marked increase in phosphorylated IκB protein levels in the colon of DSS-treated mice, which was significantly reduced following ZPE treatment, as illustrated in Figure 9D.

## 4. Discussion

Network-based pharmacological analysis revealed 260 compounds within the ZP, of which 131 were classified as active compounds (Table S2). The criteria employed for the screening of active compounds encompass three key parameters: OB (oral bioavailability), DL (drug-likeness), and Caco-2 permeability. The OB of a drug is defined as the proportion of the administered dose that is absorbed into the circulation after oral intake. The DL of a compound is assessed based on its structural properties, with Lipinski’s Rule of Five being a commonly used guideline for predicting drug-like behavior. The Caco-2 permeability of a compound is used to evaluate its ability to cross the intestinal barrier. High permeability is associated with a greater potential for oral absorption [25,26]. These 131 compounds were associated with target information, resulting in a total collection of 1121 target genes (Supplementary Material Table S5). Figure 3 shows the 37 active compounds associated with IBD. Among these active compounds, Figure 4 shows that quercetin specifically targets AKT1. In response to reactive oxygen species, the activation of NF-κB through IκB kinase is known to involve the PI3K/AKT/PTEN pathway. Consequently, this pathway is considered to have a substantial effect on CD [27].

Various claudin isoforms, such as CLDN4, form strand networks within the TJ (tight junction) plaques in the intercellular space between adjacent epithelial cells, creating selective channels surrounding the cells. Consequently, claudin dysfunction in intestinal cells can contribute to the impairment of the epithelial barrier and the development of various gastrointestinal diseases, such as IBD [28].

Deficiencies in IL-10 and its receptor, IL-10R, are observed in IBD and reflect the intestine’s susceptibility to changes in the immune system. The absence of IL-10 and IL-10R results in severe enterocolitis that manifests early in development [29]. PTGS2, commonly referred to as COX-2, is an immediate-early response gene generally not expressed in most cells. However, it is predominantly upregulated at sites of inflammation in response to inflammatory stimuli, such as cytokines including TNF-α, IL-1α/β, and IFN-γ. Abnormal mucosal immune responses to inflammation are believed to play a role in the development of chronic inflammatory conditions including IBD [30]. As shown in Figure 4, PTGS2 is targeted by 33 compounds present in the ZP, suggesting that the compounds in the ZP effectively regulate the levels of PTGS2 in the intestine. Previous studies have provided experimental validation supporting the synergistic effects of multi-compound and multi-target herbal extracts, as indicated by various analyses [31].

Several IBD-related compounds were identified, as shown in Figure 4. Additionally, 33 compounds targeting PTGS2 were identified. Quercetin targets AKT1, CASP3, CCL2, CLDN4, CRP, CXCL10, CXCL2, CXCL8, HMOX1, ICAM1, IFNG, IL10, IL1B, IL2, IL6, MPO, NFKBIA, PPARG, PTGS2, STAT1, and TNF (Figure 4). The 131 active compounds were screened by considering their ADME parameters, which indicate their potential as candidates for future drug research [32]. Several studies have documented an association between essential compounds and IBD.

Quercetin enhances the expression of TJ proteins, strengthens intestinal barrier function [33,34], and promotes intestinal cell proliferation [35]. It also maintains the regenerative capacity of intestinal mesenchymal stem cells [36]. Quercetin has crucial immune-regulatory functions [37]. It increases the proportion of Treg cells and reduces the infiltration of dendritic cells, neutrophils, and Th17 cells [34]. It also modulates the local cytokine environment and suppresses chronic intestinal inflammation [37].

Dietary fatty acids, particularly oleic acid, have been suggested to be involved in the onset of UC. AA (arachidonic acid) serves as a precursor for inflammatory cytokines, such as leukotriene B4 and prostaglandin E2. Oleic acid competitively inhibits AA metabolism [38]. The compounds exhibited interactions with an average of 19.88 target genes, highlighting the multi-compound–multi-target nature of herbal medicines (Figure 2). It is anticipated that ZP may exhibit therapeutic effects in the management of IBD due to the synergistic properties of the diverse compounds found in ZP. Based on these comprehensive findings, it can be concluded that ZP has the potential to treat IBD and that its therapeutic effects are attributed to the inhibition of immune cell infiltration. Furthermore, the mechanism was suggested to be associated with the interactions between 59 key components, such as quercetin, and 38 IBD-related genes, including AKT1, CLDN4, IL-10, and PTGS2.

Using network pharmacology analysis, we performed docking studies on PTGS2, which formed the most extensive network with active compounds of ZP among the genes related to IBD. The findings demonstrated that PTGS2, along with 15 of these IBD-related compounds, displayed potential binding activity, with binding energies below −7 kcal/mol. These compounds are promising candidates for future drug research (Table 2, Figure 5).

Through an investigation using DSS-induced mice, we aimed to assess the beneficial effects of ZPE on DSS-induced intestinal leakage and liver damage because research on the effects of ZPE on these aspects is limited. The potential of ZPE to provide relief from colitis and liver injury was also explored. In order to assess the antioxidant and anti-inflammatory effects of ZPE on IBD, a model of colitis induced via DSS was employed using a laboratory mouse. The results obtained from this study provide compelling evidence that ZPE effectively mitigates weight loss, DAI score, occult/gross bleeding, and diarrhea in mice with DSS-induced colitis compared to no treatment in the DSS group (Figure 6).

Previous studies have consistently revealed a strong connection between the inflammation observed in IBD and the resultant damage to multiple organs, including the liver, via the gut–liver axis. Inflammation and liver damage can occur in individuals with IBD. One study reported that up to 50% of patients with IBD experienced liver dysfunction and biliary disorders [39]. However, the beneficial effects of ZPE on liver damage resulting from intestinal structural and barrier dysfunction have not yet been investigated. According to our data, there were no significant changes in liver weight or markers of liver damage, such as abnormal H&E staining histopathology, in DSS-treated mice with colitis, and ZPE treatment did not affect these markers of liver damage (Figure 7A,B).

The pro-inflammatory enzyme PTGS2 is upregulated in the colon of patients with IBD as well as in a DSS-induced colitis model. Furthermore, PTGS2 was identified as a pivotal target, exhibiting the highest correlation with the network of genes associated with IBD and compounds from ZP (Figure 4). PTGS2 was also employed as the primary target in docking simulations to investigate active compound candidates of ZP (Figure 5). And, PARP activation, which is associated with apoptosis, can act as a target in the iNOS/PTGS2/NF-κB-associated inflammatory pathway in both the intestine and liver. Activation is commonly observed under conditions of increased oxidative stress. Elevated oxidative/nitrosative stress and inflammatory markers in the liver were observed only with iNOS, particularly in response to DSS exposure. ZPE supplementation effectively mitigated these effects (Figure 7C). However, aside from these markers, there were no noteworthy disparities in the expression levels of liver regeneration markers, such as Cyclin D, cdk2, cdk4, and eNOS, between DSS-exposed colitis mice and those subjected to ZPE treatment (Figure 7D).

Among the two major forms of IBD, Crohn’s disease can affect any part of the gastrointestinal tract, including the colon and small intestine, while ulcerative colitis is generally confined to the colon but can extend to the terminal ileum [40,41], leading to backwash ileitis. Given this association between IBD and the small intestine, we examined the effects on the small intestine in a DSS-induced colitis model [42].

There were no significant variations in the protein expression levels of key markers associated with inflammation, oxidative stress, and nitration in the small intestines of mice with DSS-induced colitis. These included PTGS2, iNOS, and nitrated proteins. Furthermore, ZPE intake had no significant effect on these markers (Figure 8C). NF-κB, a transcription factor, is predominantly activated via phosphorylation of inhibitory proteins such as IκB, a process mediated by IKK (IκB kinase). This activation is crucial for regulating processes including inflammation, immune responses, cell proliferation, and apoptosis [43]. Nuclear translocation of NF-κB is a crucial regulator of inflammation and is prominently activated in both the DSS-induced colitis mouse model and in individuals with IBD. However, in the small intestines of mice with DSS-induced colitis, there was no activation observed in the NF-κB signaling pathway. In addition, no discernible differences were observed with ZPE treatment in this pathway (Figure 8D). Moreover, ZPE did not induce any significant alterations in the diminished length or integrity of the intestinal barrier in the IBD mouse model (Figure 8A,B).

Mice with DSS-induced colitis exhibited a reduction in colon length compared to healthy mice. However, this decrease was reversed by ZPE treatment (Figure 9A). Additionally, histological analysis of the colonic epithelium demonstrated that the ulceration, inflammation, and crypt loss observed in DSS-treated mice were mitigated by ZPE treatment (Figure 9B). Significant upregulation of oxidative stress and inflammatory markers, including PTGS2, iNOS, and nitrated proteins, was observed in the colons of mice with DSS-induced colitis. However, the administration of ZPE significantly diminished the elevation of these proteins in the colon, demonstrating the protective effect of ZPE in mice with DSS-induced colitis (Figure 9C). In particular, PTGS2 is a key target identified through network pharmacological analysis of IBD and ZP (Figure 4 and Figure 5). This result demonstrates a strong correlation between network pharmacological analysis and empirical experimental outcomes. The activation of the NF-κB signaling pathway was indicated by a significant increase in IκB activation in the colons of mice with DSS-induced colitis. However, following treatment with ZPE, the phosphorylation of IκB protein triggered by DSS was diminished in the colon (Figure 9D). In summary, the treatment of ZPE in mice exposed to DSS effectively prevented the activation of the inflammatory pathway and mitigated the state of oxidative stress by inhibiting the pathway of NF-κB signaling.

In our study, the DSS-induced colitis mouse model caused damage by targeting the colon rather than the liver and small intestine. Furthermore, our findings indicate that the antioxidant and anti-inflammatory effects of ZPE can mitigate colon damage induced by DSS-induced colitis. Notably, PTGS2, which was identified through network pharmacological analysis as a key target for ZP in DSS-induced colitis, was also confirmed by experimental evidence. This highlights the potential of network pharmacological analysis to complement empirical experimental results. Therefore, it is expected that ZP can serve as a safe dietary supplement to support patients with IBD.

## 5. Conclusions

Our study explored the potential of ZP using a network-based pharmacological analysis. This analysis revealed the involvement of 131 active compounds and 1121 genes associated with ZP. Moreover, the study proposed the possibility of antioxidant and anti-inflammatory mechanisms, including interactions between 59 active compounds, such as quercetin, and 38 IBD-related genes, such as PTGS2. Additionally, our animal study demonstrated the protective effects of ZPE dietary intervention in the colon of DSS-exposed mice through iNOS, NF-κB, IκB, nitrated proteins, and specifically PTGS2, the key target in network pharmacology. These findings highlight the potential of ZP as a therapeutic agent for IBD.

## Figures and Tables

**Table 1:** Active compounds of *Zanthoxyli Pericarpium*.

No.	Molecule Name	Pubchem ID
1	(−)-Citronellal	443157
2	(−)-*N*-acetylanonaine	78411088
3	(+)-Ledol	92812
4	(+)-Pinoresinol O-B-D-glucopyranoside	486614
5	(+)-Pinoresinol-3-hydroxy-4-methyl-4-pentenyl ether	-
6	(+)-trans-piperitenol	85568
7	(1R,4R)-4-isopropyl-1-methyl-2-cyclohexen-1-ol	-
8	(1S,3R,5S)-6,6-dimethyl-2-methylene-3-norpinanol	1201530
9	(1S,4S)-1-isopropyl-4-methyl-3-bicyclo[3.1.0]hexanone	-
10	(2E,4E,8E,10E,12E)-*N*-Isobutyl-2,4,8,10,12-tetradecapenta-enamide	5318518
11	(2R-cis)-1,2,3,4,4a,5,6,7-Octahydro-alpha	-
12	(2S)-3-methoxypropane-1,2-diol	156179
13	(6R)-6-isopropyl-3-methyl-1-cyclohex-2-enone	107561
14	(6R,7R)-Caryophyllene	-
15	(E)-linalool oxide acetate pyr	6427501
16	(e)-sabinene,hydrate	6326181
17	(s)-carvone	16724
18	1-alpha-Terpinyl acetate	11469649
19	1H-Cycloprop(e)azulen-7-ol, decahydro-1,1,7-trimethyl-4-methylene-, (1aR-(1aalpha,4aalpha,7beta,7abeta,7balpha))-	97032059
20	1-heptanol	8129
21	1-hexanol	8103
22	1-Methoxy-4-(1-propenyl)benzene	637563
23	1-methoxy-4-(2-propenyl)-benzene	8815
24	1-Methoxyrutaecarpine	-
25	1-nonanol	8914
26	2,3,13-trimethoxy-5,11a-dihydro[1,3]dioxolo[4′,5′:4,5]-benzo[c]phenanthridine	-
27	2-[(1R,3S,4S)-3-isopropenyl-4-methyl-4-vinylcyclohexyl]propan-2-ol	92138
28	2-Propanone, 1, 3-dihydroxy-	670
29	3,4-Dimethoxy-3′,4′-methylenedioxy-7,9′-epoxylignan-9-ol	-
30	4-(1-Methylethyl)-1-cyclohexene-1-carboxaldehyde	89488
31	4-Isopropylcyclohexanone	79488
32	4-methyl-1-heptanol	109451
33	4-methyl-1-isopropyl-3-cyclohexen-1-ol	11230
34	5-methoxydictamnine	-
35	6-methoxy[1,3]dioxolo[4′,5′:4,5]benzo[c][1,3]dioxolo[4,5-j]phenanthridine	-
36	6-methyl-1-heptanol	15450
37	7-(5′,6′-Dihydroxy-3′,7′-dimethylocta-2′,7′-dienyloxy)-coumarin	-
38	7,9-Dimethoxy-2,3-methylendioxybenzophenanthridine	-
39	8-(2-isopentenyloxy)-4,7-dimethoxyfuro[2,3-b]quinoline	13970973
40	8-hydroxy-4,7-dimethoxy-furanoquinoline	371227
41	8-methoxyisodecarine	136854509
42	8-Methyl-5-isopropyl-6,8-nonadiene-2-one	5319691
43	Acetoxyauraptene	-
44	Ailanthamide	25195168
45	alpha-elemol	92138
46	alpha-terpinol	442501
47	Anizol	7519
48	Ascaridole	12308625
49	Acetone	180
50	benzaldehyde,4-(1-methylethyl)	326
51	benzeneacetic acid, methyl ester	87574380
52	Berberime	2353
53	Berberine	2353
54	beta-Cubebene	93081
55	beta-Gurjunene	6450812
56	beta-sitosterol	222284
57	beta-thujone	261491
58	BOX	243
59	Butenone	6570
60	Capsaicin	1548943
61	Chelerythrine	5351594
62	cis-9-hexadecenoic acid	445638
63	cis-linalol pyranoxide	6428574
64	cis-p-2-menthen- 1-ol	141999
65	cis-Pinene hydrate	1268143
66	Cis-p-mentha-2,8-dien-1-ol	111274
67	cis-sabinene hydrate	62367
68	Citronellol	8842
69	CLOVENE	10102
70	Collinin	5316012
71	Cuminol	325
72	D-Borneol	6552009
73	Dehydro-γ-sanshool	-
74	Dihydrobungeanool	101936592
75	Dihydrochelerythrine	485077
76	Diosmetin	5281612
77	Estriol	16757678
78	Eucalyptol	34365085
79	Fagaridine	177893
80	gallic acid	370
81	Geranial	638011
82	Haplopine	5281846
83	heptanoic acid;heptylic acid;enanthylic acid	8094
84	Herniarin	10748
85	hydroxy-beta-sanshool	10220912
86	hydroxy-Epsilon-sanshool	46870578
87	hydroxyl-alpha-sanshool	10084135
88	Isodecarine	135844948
89	Isofagaridine	177893
90	Isopiperitenone	79036
91	Isopulegol	170833
92	Kokusaginin	10227
93	lanyuamide V	101153415
94	lanyuamide VI	101153416
95	Lemairamide	9468490
96	Lemairamin	1376042
97	linalyl anthranilate	23535
98	Majudin	2355
99	Meso-2,3-bis(3,4,5-trimethoxybenzyl)-1,4-butanediol	-
100	methyl 4-methylvalerate	17008
101	Methyleugenol	7127
102	Mnk	8163
103	myrcene epoxide	122371
104	nerohdyl acetate	25021983
105	Nerol acetate	1549025
106	Nitidine	4501
107	*N*-nornitidine	296524
108	oleic acid	445639
109	o-methylacetophenone	11340
110	Oxychelerythrine	147279
111	p-1,8-menthadienyl-7 acetate	-
112	quercetin	5280343
113	rhoifoline A	5282150
114	Sabinene hydrate	62367
115	sanshool	6440935
116	Schinicoumarin	5321163
117	Skimmetin	5281426
118	Skimmianin	6760
119	Skimmianine	6760
120	spathulenol	9794468
121	suberosin	68486
122	tamgeretin	68077
123	timuramide C	71524339
124	T-Muurolol	3084331
125	trans-4-isopropyl-1-methylcyclohex-2-en-1-ol	5319367
126	trans-limonene,oxide	449290
127	Trans-p-mentha-2,8-dien-1-ol	12618691
128	WLN: VH6	8130
129	Zanthobungeanine	5315422
130	zoomaric acid	445638
131	α-cadinol	10398656

**Table 2:** Binding affinities for interaction proteins (PTGS2) and 32 active compounds.

Compound	Target (PDB * ID)	Affinity (kcal/mol)
Diosmetin	PTGS2 (5ikq)	−9.677
Quercetin	PTGS2 (5ikq)	−9.562
Nitidine	PTGS2 (5ikq)	−9.553
1-Methoxyrutaecarpine	PTGS2 (5ikq)	−9.333
Oxychelerythrine	PTGS2 (5ikq)	−9.227
Berberine	PTGS2 (5ikq)	−9.029
Dihydrochelerythrine	PTGS2 (5ikq)	−8.803
beta-sitosterol	PTGS2 (5ikq)	−8.538
Tamgeretin	PTGS2 (5ikq)	−8.321
Eucalyptol	PTGS2 (5ikq)	−8.315
Suberosin	PTGS2 (5ikq)	−7.733
Skimmetin	PTGS2 (5ikq)	−7.342
Haplopine	PTGS2 (5ikq)	−7.193
Herniarin	PTGS2 (5ikq)	−7.125
beta-Cubebene	PTGS2 (5ikq)	−7.004
Capsaicin	PTGS2 (5ikq)	−6.962
oleic acid	PTGS2 (5ikq)	−6.877
linalyl anthranilate	PTGS2 (5ikq)	−6.834
CLOVENE	PTGS2 (5ikq)	−6.81
gallic acid	PTGS2 (5ikq)	−6.303
1-alpha-Terpinyl acetate	PTGS2 (5ikq)	−6.046
1-Methoxy-4-(1-propenyl)benzene	PTGS2 (5ikq)	−6.003
Methyleugenol	PTGS2 (5ikq)	−5.878
1-methoxy-4-(2-propenyl)-benzene	PTGS2 (5ikq)	−5.869
BOX	PTGS2 (5ikq)	−5.614
nerohdyl acetate	PTGS2 (5ikq)	−5.583
zoomaric acid	PTGS2 (5ikq)	−5.57
(E)-linalool oxide acetate pyr	PTGS2 (5ikq)	−5.421
Nerol acetate	PTGS2 (5ikq)	−5.203
Citronellol	PTGS2 (5ikq)	−5.177
cis-9-hexadecenoic acid	PTGS2 (5ikq)	−4.609
Acetone	PTGS2 (5ikq)	−3.182

* PDB means Protein Data Bank.

## Data Availability

The data presented in this study are available from the corresponding author upon request. The data are not publicly available due to privacy and ethical restrictions.

## Supplementary Materials

The following supporting information can be downloaded at: https://www.mdpi.com/article/10.3390/nu16203521/s1. Figure S1: UPLC profiles of 3 major compounds identified in ZP; Table S1: The analysis condition of Bergapten, Auraptene, and Xanthoxylin; Table S2: Three marker compounds in ZP based on UPLC results; Table S3: Scoring system to calculate the disease activity index (DAI); Table S4: Total compounds of *Zanthoxyli Pericarpium*; Table S5: Target genes of *Zanthoxyli Pericarpium*; Table S6: 100 Disease-related genes.
