# Supplementary material for: Therapeutic Effects of Zanthoxyli Pericarpium on Intestinal Inflammation and Network Pharmacological Mechanism Analysis in a Dextran Sodium Sulfate-Induced Colitis Mouse Model
Source: Nutrients. 2024 Oct 17;16(20):3521. doi: 10.3390/nu16203521 (PMC11510417; doi:10.3390/nu16203521)
Supplement: Supplementary file 1 [file nutrients-16-03521-s001.zip › Figure S1 UPLC profiles of 3 major compounds.pdf]

Figure S1. UPLC profiles of 3 major compounds identified in ZP.

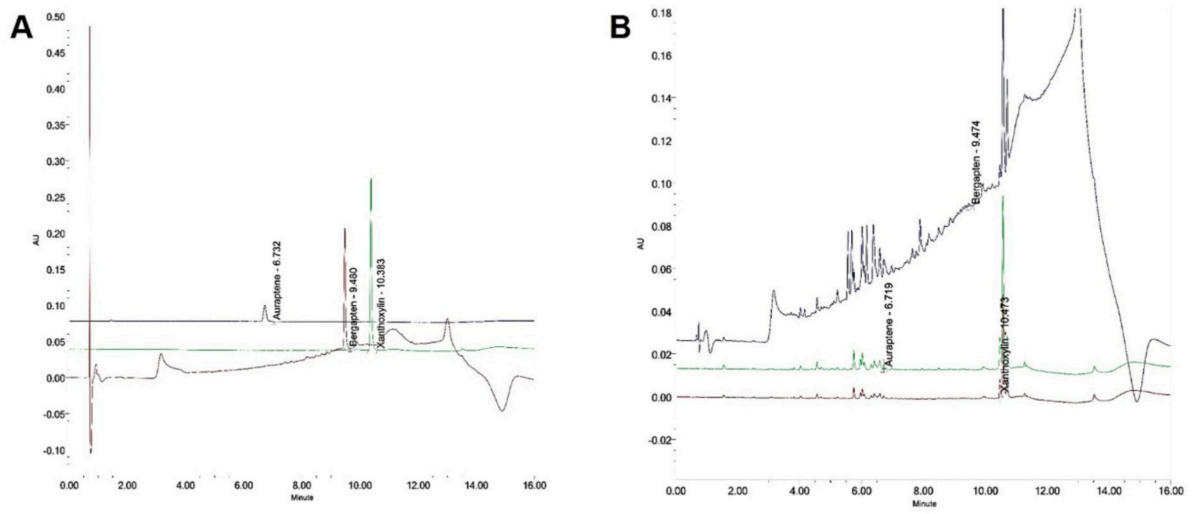

Figure S1. UPLC profiles of 3 major compounds identified in ZP. (A) UPLC profile of the commercial standard compounds. (B) UPLC profile of 3 major compounds in ZP. Chromatograms were obtained at 280 nmol/L (Bergapten) and 380 nmol/L (Auraptene and Xanthoxylin).
