# Supplementary material for: Therapeutic Effects of Zanthoxyli Pericarpium on Intestinal Inflammation and Network Pharmacological Mechanism Analysis in a Dextran Sodium Sulfate-Induced Colitis Mouse Model
Source: Nutrients. 2024 Oct 17;16(20):3521. doi: 10.3390/nu16203521 (PMC11510417; doi:10.3390/nu16203521)
Supplement: Supplementary file 1 [file nutrients-16-03521-s001.zip › Table S1 The analysis condition.pdf]

Table S1. The analysis condition of Bergapten, Auraptene and Xanthoxylin.

Time(minute)	0.1% FA / water (%)	0.1% FA / acetonitrile (%)	Flow rate (ml/minute)
0	98	2	0.40
1.0	98	2	0.40
2.0	90	10	0.40
3.0	80	20	0.40
5.0	70	30	0.40
6.0	60	40	0.40
8.0	50	50	0.40
9.0	20	80	0.40
10.0	10	90	0.40
12.0	2	98	0.40
14.0	98	2	0.40
16.0	98	2	0.40
