# Supplementary material for: Therapeutic Effects of Zanthoxyli Pericarpium on Intestinal Inflammation and Network Pharmacological Mechanism Analysis in a Dextran Sodium Sulfate-Induced Colitis Mouse Model
Source: Nutrients. 2024 Oct 17;16(20):3521. doi: 10.3390/nu16203521 (PMC11510417; doi:10.3390/nu16203521)
Supplement: Supplementary file 1 [file nutrients-16-03521-s001.zip › Table S2 3 marker compounds in ZP by UPLC.pdf]

Table S2. 3 marker compounds in ZP by UPLC

	unit:mg/kg
**Auraptene**	**2.182±0.067**
**Xanthoxylin**	**1.185±0.024**
**Bergapten**	**0.481±0.005**
