# Supplementary material for: Therapeutic Effects of Zanthoxyli Pericarpium on Intestinal Inflammation and Network Pharmacological Mechanism Analysis in a Dextran Sodium Sulfate-Induced Colitis Mouse Model
Source: Nutrients. 2024 Oct 17;16(20):3521. doi: 10.3390/nu16203521 (PMC11510417; doi:10.3390/nu16203521)
Supplement: Supplementary file 1 [file nutrients-16-03521-s001.zip › Table S3 Scoring system to calculate.pdf]

Table S3. Scoring system to calculate the disease activity index (DAI). The DAI value is obtained by averaging the scores for weight loss, stool consistency, and presence of blood in feces.

Score	Weight loss	Stool consistency	Visible blood feces
**0**	**None**	**Normal**	**None**
**1**	**1~5%**		
**2**	**6~10%**	**Loose**	**Slight bleeding**
**3**	**11~20%**		
**4**	**<20%**	**Diarrhea**	**Gross bleeding**
