# Supplementary material for: Therapeutic Effects of Zanthoxyli Pericarpium on Intestinal Inflammation and Network Pharmacological Mechanism Analysis in a Dextran Sodium Sulfate-Induced Colitis Mouse Model
Source: Nutrients. 2024 Oct 17;16(20):3521. doi: 10.3390/nu16203521 (PMC11510417; doi:10.3390/nu16203521)
Supplement: Supplementary file 1 [file nutrients-16-03521-s001.zip › Table S4 Total compounds of Zanthoxyli Pericarpium.pdf]

Table S4. Total compounds of Zanthoxyli Pericarpium

No.	Molecule Name	Source		
		ETCM2	TCMBank	TCMSP
1	(-)-alpha-Pinene			O
2	(-)-beta-Phellandrene			O
3	(-)-bornyl,acetate	O		
4	(-)-Citronellal		O	
5	(-)-Comphene			O
6	(-)-N-acetylanonaine	O		
7	(-)-nopinene			O
8	(-)-spathulenol	O		
9	()-Terpinen-4-ol			O
10	(-)-trans-isopiperitenol	O		
11	(-)-β-caryophyllene	O		
12	(+)-Beta-Caryophyllene	O		
13	(+)-cis-nerolidol	O		
14	(+)-Ledol		O	O
15	(+)-Pinoresinol O-B-D-glucopyranoside	O		
16	(+)-Pinoresinol-3,3-dimethylallyl ether	O		
17	(+)-Pinoresinol-3-hydroxy-4-methyl-4-pentenyl ether	O		
18	(+)-Pinoresinol-di-3,3-dimethylallyl ether	O		
19	(+)-trans-Carveol	O		
20	(+)-trans-piperitenol	O		
21	(±)-tembetarine	O		
22	(±)-α-terpinyl acetate	O		
23	(1R,4R)-4-isopropyl-1-methyl-2-cyclohexen-1-ol	O		
24	(1S,2S)-2-isopropenyl-4-isopropylidene-1-methyl-1-vinylcyclohexane			O
25	(1S,3R,5S)-6,6-dimethyl-2-methylene-3-norpinanol	O		
26	(1S,4S)-1-isopropyl-4-methyl-3-bicyclo[3.1.0]hexanone	O		
27	(1S,5S)-1-isopropyl-4-methylenebicyclo[3.1.0]hexane			O
28	(2e,4 e,8z,11z)-2'-hydroxy-n-isobutyl-2,4,8,11-tetradecatetraenamide		O	
29	(2E,4E,8E,10E,12E)-N-Isobutyl-2,4,8,10,12-tetradecapenta-enamide	O		
30	(2R-cis)-1,2,3,4,4a,5,6,7-Octahydro-alpha	O		
31	(2S)-3-methoxypropane-1,2-diol		O	O
32	(6R)-6-isopropyl-3-methyl-1-cyclohex-2-enone		O	O
33	(6R,7R)-Caryophyllene	O		
34	(E)-linalool oxide acetate pyr		O	O
35	(e)-sabinene,hydrate	O		
36	(L)-alpha-Terpineol			O
37	(R)-(-)-alpha-Phellandrene			O
38	(R)-linalool			O
39	(s)-carvone	O		
40	.alpha.-Carene			O
41	[(3S)-3,7-dimethyloct-6-enyl] acetate			O

No.	Molecule Name	Source		
		ETCM2	TCMBank	TCMSP
42	1,2,3,4,4a,5,6,8a-octahydro-7-methyl-4-methylene-1-(1-methyl)-naphthalene	O		
43	1,2,3,4,4a,5,6,8a-Octahydro-7-methyl-4-methylene-1-(1-methylethyl)-naphthalene	O		
44	1,8-cineole			O
45	10-epi-gamma-eudesmol	O		
46	1-alpha-Terpinyl acetate		O	O
47	1H-Cycloprop(e)azulen-7-ol, decahydro-1,1,7-trimethyl-4-methylene-, (1aR-(1aalpha,4aalpha,7beta,7abeta,7balpha))-		O	
48	1-heptanol	O		
49	1-hexanol	O		
50	1-Isopropyl-4-methylene-7-methyl-1,2,3,4,4a,5,6,8a-octahydro-naphthalene	O		
51	1-Methoxy-4-(1-propenyl)benzene	O		
52	1-methoxy-4-(2-propenyl)-benzene		O	
53	1-Methoxyrutaecarpine	O		
54	1-nonanol	O		
55	2,3,13-trimethoxy-5,11a-dihydro[1,3]dioxolo[4',5':4,5]-benzo[c]phenanthridine	O		
56	2,6-dimethylheptanal	O		
57	2-[(1R,3S,4S)-3-isopropenyl-4-methyl-4-vinylcyclohexyl]propan-2-ol	O		
58	2-methoxy-4-propenyl phenol		O	
59	2-Methyl-5-(1-methylethenyl)-2-cyclohexen-1-ol	O		
60	2-Propanone, 1, 3-dihydroxy-		O	
61	2-tridecanone	O		
62	3,4,5-trihydroxybenzoic acid			O
63	3,4-Dimethoxy-3',4'-methylenedioxy-7,9'-epoxylignan-9-ol	O		
64	3-Methyl-6-(1-methylethyl)-2-cyclohexen-1-ol	O		
65	3-methylhexane			O
66	3-methylpentadecane	O		
67	3-nonanone	O		
68	3-Oxo-skimmiarepin	O		
69	4-(1-Methylethyl)-1-cyclohexene-1-carboxaldehyde	O		
70	4-(1-Methylethyl)-2-cyclohexen-1-one	O		
71	4-isopropylcyclohex-2-en-1-one			O
72	4-Isopropylcyclohexanone	O		
73	4-Methyl-1-(1-methylethyl)-3-cyclohexen-1-ol	O		
74	4-methyl-1-heptanol	O		
75	4-methyl-1-isopropyl-3-cyclohexen-1-ol		O	
76	4-terpinenol	O		
77	4-terpineol	O		
78	5-methoxydictamnine	O		
79	6-methoxy[1,3]dioxolo[4',5':4,5]benzo[c][1,3]dioxolo[4,5-j]phenanthridine	O		
80	6-methyl-1-heptanol	O		
81	6-methylheptanal	O		

No.	Molecule Name	Source		
		ETCM2	TCMBank	TCMSP
82	7-(5',6'-Dihydroxy-3',7'-dimethylocta-2',7'-dienyloxy)-coumarin	O		
83	7,9-Dimethoxy-2,3-methylendioxybenzophenanthridine	O		
84	8-(2-isopentenyloxy)-4,7-dimethoxyfuro[2,3-b]quinoline	O		
85	8-hydroxy-4,7-dimethoxy-furanoquinoline	O		
86	8-methoxyisodecarine	O		
87	8-Methyl-5-isopropyl-6,8-monadiene-2-one	O		
88	8-Methyl-5-isopropyl-6,8-nonadiene-2-one	O	O	
89	Acetoxyauraptene	O		
90	ailanthamide	O		
91	alpha-Cadinol	O		
92	alpha-cis-bergamotene	O		
93	alpha-elemol	O		
94	alpha-humulene			O
95	Alpha-muurolene	O		
96	alpha-terpinol		O	
97	alpha-Terpinyl acetate	O		
98	alpha-Thujone	O		
99	anethole			O
100	Anizol		O	O
101	ascaridole		O	O
102	Auraptene	O		
103	Ayapanin			O
104	Azeton		O	O
105	benzaldehyde,4-(1-methylethyl)		O	
106	benzeneacetic acid, methyl ester	O		
107	berberime		O	
108	berberine		O	
109	beta-caryophyllene			O
110	beta-Cubebene			O
111	beta-elemene			O
112	beta-Gurjunene			O
113	beta-Selinene			O
114	beta-sitosterol			O
115	beta-thujone	O		
116	Bicyclogermacrene	O		
117	b-Methylaseculetin	O		
118	BOX		O	O
119	Bungeanool	O		
120	Butenone		O	O
121	CADINENE			O
122	Cadinol	O		
123	capsaicin		O	
124	Car-3-ene			O

No.	Molecule Name	Source		
		ETCM2	TCMBank	TCMSP
125	caryophyllene oxide	O		
126	cedrelanol	O		
127	Chelerythrine		O	
128	cineole			O
129	cis-9-hexadecenoic acid		O	
130	cis-Anethol			O
131	cis-carveyl acetate	O		
132	cis-linalol pyranoxide		O	O
133	cis-nerolidol			O
134	cis-p-2-menthen- 1-ol	O		
135	cis-Pinene hydrate		O	O
136	Cis-p-mentha-2,8-dien-1-ol	O		
137	cis-sabinene hydrate	O		
138	citronellol		O	
139	CLOVENE			O
140	Collinin	O		
141	cryptone		O	
142	cuminal			O
143	Cuminol		O	O
144	cumulene		O	
145	Cymol			O
146	D-Borneol	O		
147	Dehydro-γ-sanshool	O		
148	delta-amorphene			O
149	D-Germacrene	O		
150	Dihydrobungeanool	O		
151	Dihydrochelerythrine		O	
152	Diosmetin		O	O
153	Diosmin			O
154	Dymel A			O
155	Estriol		O	
156	eucalyptol		O	
157	Eudesma-4(14),11-diene	O		
158	eugenol			O
159	fagaridine	O		
160	gallic acid		O	
161	gamma-Cadinene	O		
162	geranial		O	
163	geraniol			O
164	geraniol acetate		O	
165	germacrene	O		
166	Haplopine		O	O
167	hazaleamide	O		

No.	Molecule Name	Source		
		ETCM2	TCMBank	TCMSP
168	heptanoic acid			O
169	heptanoic acid;heptylic acid;enanthylic acid		O	
170	heptyl acetate	O		
171	herniarin		O	
172	HEX			O
173	Hexyl acetate	O		
174	hydroxy-beta-sanshool	O		
175	hydroxy-Epsilon-sanshool	O		
176	hydroxyl-alpha-sanshool	O		
177	Hyperin			O
178	Isodecanal	O		
179	isodecarine	O		
180	Isofagaridine	O		
181	Isopiperitenone	O		
182	Isopulegol	O		
183	Kokusaginin			O
184	kokusaginine		O	
185	lanyuamide IV	O		
186	lanyuamide V	O		
187	lanyuamide VI	O		
188	L-Bornyl acetate			O
189	Lemairamide	O		
190	Lemairamin	O		
191	limonene	O		
192	linalyl anthranilate		O	O
193	L-Limonen			O
194	Majudin		O	
195	Meso-2,3-bis(3,4,5-trimethoxybenzyl)-1,4-butanediol	O		
196	methyl 4-methylvalerate	O		
197	methyl isothiocyanate		O	
198	Methyleugenol		O	O
199	Mnk		O	O
200	Moslene			O
201	Myrcene			O
202	myrcene epoxide	O		
203	Myrtenyl acetate	O		
204	N-(4-methoxyphenethyl)benzamide	O		
205	n-acetylanonaire		O	
206	Naphthalene, 1,2,3,4,4a,5,6,8a-octahydro-7-methyl-4-methylene-1-(1-methylethyl)-, (1alpha,4abeta,8aalpha)-			O
207	Neoiso-isopulegol	O		
208	nerohdyl acetate			O
209	Nerol acetate		O	

No.	Molecule Name	Source		
		ETCM2	TCMBank	TCMSP
210	NERYLACETATE			O
211	nitidine		O	
212	N-nornitidine	O		
213	o-Acetyltoluene			O
214	o-Cymol			O
215	oleic acid			O
216	o-methylacetophenone		O	
217	orcinol glucoside	O		
218	Otan			O
219	Oxychelerythrine		O	
220	p-1,8-menthadienyl-7 acetate	O		
221	palmitic acid			O
222	Perillen		O	
223	p-mentha-1,8(10)-dien-9-ol	O		
224	quercetin		O	O
225	rhoifoline A	O		
226	Sabinene hydrate	O		
227	sanshool		O	O
228	Schinicoumarin	O		
229	Schinifoline		O	
230	skimimanine		O	
231	Skimmetin		O	O
232	Skimmianin			O
233	Skimmianine	O		
234	solanone		O	
235	spathulenol			O
236	suberosin		O	O
237	Tamarixetin 3-O-β-D-glucopyranoside 7-O-β-D-glucopyranoside	O		
238	tamgeretin		O	
239	tau-cadinol	O		
240	tau-muurolol	O		
241	T-BUTYLBENZENE			O
242	Terpilene			O
243	Terragon			O
244	timuramide C	O		
245	T-Muurolol	O		O
246	Toluene	O		
247	trans-4-isopropyl-1-methylcyclohex-2-en-1-ol	O		
248	trans-limonene,oxide	O		
249	trans-myrtanol	O		
250	Trans-p-mentha-2,8-dien-1-ol	O		
251	trans-sabinene hydrate acetate	O		
252	tricyclene			O

No.	Molecule Name	Source		
		ETCM2	TCMBank	TCMSP
253	Umbelliprenin	O		
254	WLN: VH6		O	
255	Zanthobungeanine	O	O	
256	zanthosimuline		O	
257	zanthoxylene		O	
258	ZINC02040970			O
259	zoomaric acid			O
260	α-cadinol		O	
