# Supplementary material for: Therapeutic Effects of Zanthoxyli Pericarpium on Intestinal Inflammation and Network Pharmacological Mechanism Analysis in a Dextran Sodium Sulfate-Induced Colitis Mouse Model
Source: Nutrients. 2024 Oct 17;16(20):3521. doi: 10.3390/nu16203521 (PMC11510417; doi:10.3390/nu16203521)
Supplement: Supplementary file 1 [file nutrients-16-03521-s001.zip › Table S5 Target genes of Zanthoxyli Pericarpium.pdf]

Table S5. Target genes of *Zanthoxyli Pericarpium*

Target name	Gene Name	UniProt ID
11-beta-hydroxysteroid dehydrogenase 1	HSD11B1	P28845
17-beta-hydroxysteroid dehydrogenase type 3	HSD17B3	P37058
26S proteasome non-ATPase regulatory subunit 3	PSMD3	O43242
3-hydroxy-3-methylglutaryl-CoA reductase	HMGCR	P04035
3-hydroxy-3-methylglutaryl-coenzyme A reductase	HMGCR	P04035
3-oxo-5-alpha-steroid 4-dehydrogenase 2	SRD5A2	P31213
4-aminobutyrate aminotransferase	ABAT	P80404
5'-aminolevulinate synthase 2	ALAS2	P22557
5-hydroxytryptamine 2A receptor	HTR2A	P28223
5-hydroxytryptamine receptor 1D	HTR1D	P28221
5-hydroxytryptamine receptor 2B	HTR2B	P41595
5-hydroxytryptamine receptor 2B	HTR2B	P41595
5-hydroxytryptamine receptor 4	HTR4	Q13639
5-hydroxytryptamine receptor 6	HTR6	P50406
5-hydroxytryptamine receptor 7	HTR7	P34969
5-methyltetrahydrofolate-homocysteine methyltransferase reductase	MTRR	Q9UBK8
5-phosphohydroxy-L-lysine phospho-lyase	PHYKPL	Q8IUZ5
72 kDa type IV collagenase	MMP2	P08253
78 kDa glucose-regulated protein	HEL-S-89n	V9HWB4
ABL proto-oncogene 1, non-receptor tyrosine kinase	ABL1	P00519
ABL proto-oncogene 2, non-receptor tyrosine kinase	ABL2	P42684
Acetylcholinesterase	ACHE	P22303
acetyl-CoA acetyltransferase 2	ACAT2	Q9BWD1
acetyl-CoA acyltransferase 2	ACAA2	P42765

Target name	Gene Name	UniProt ID
Acetyl-CoA carboxylase 1	ACACA	Q13085
acid phosphatase 1	ACP1	P24666
acid phosphatase 3	ACP3	P15309
acid phosphatase 4	ACP4	Q9BZG2
acid phosphatase 5, tartrate resistant	ACP5	P13686
acrosin	ACR	P10323
activating transcription factor 3	ATF3	P18847
activating transcription factor 4	ATF4	P18848
Activator of 90 kDa heat shock protein ATPase homolog 1	AHSA1	O95433
acyl-CoA dehydrogenase short/branched chain	ACADSB	P45954
Acyl-CoA desaturase	FADS2	O95864
acyl-CoA oxidase 1	ACOX1	Q15067
acyl-CoA synthetase long chain family member 3	ACSL3	O95573
adducin 1	ADD1	P35611
adducin 2	ADD2	P35612
adducin 3	ADD3	Q9UEY8
adenine phosphoribosyltransferase	APRT	P07741
adenosylhomocysteinase	AHCY	P23526
adenylosuccinate synthase 1	ADSS1	Q8N142
adiponectin, C1Q and collagen domain containing	ADIPOQ	Q15848
ADP ribosylation factor 1	ARF1	P84077
adrenoceptor alpha 1A	ADRA1A	P35348
adrenoceptor alpha 1B	ADRA1B	P35368
adrenoceptor alpha 1D	ADRA1D	P25100
adrenoceptor alpha 2A	ADRA2A	P08913

Target name	Gene Name	UniProt ID
adrenoceptor alpha 2B	ADRA2B	P18089
adrenoceptor alpha 2C	ADRA2C	P18825
adrenoceptor beta 1	ADRB1	P08588
AKT serine/threonine kinase 1	AKT1	P31749
alanine--glyoxylate aminotransferase 2	AGXT2	Q9BYV1
alanine--glyoxylate and serine--pyruvate aminotransferase	AGXT	P21549
alanyl-tRNA synthetase 1	AARS1	P49588
alanyl-tRNA synthetase 2, mitochondrial	AARS2	Q5JTZ9
albumin	ALB	P02768
Alcohol dehydrogenase 1A	ADH1A	P07327
Alcohol dehydrogenase 1B	ADH1B	P00325
alcohol dehydrogenase 1B (class I), beta polypeptide	ADH1B	P00325
Alcohol dehydrogenase 1C	ADH1C	P00326
alcohol dehydrogenase 7 (class IV), mu or sigma polypeptide	ADH7	P40394
aldehyde dehydrogenase 1 family member A1	ALDH1A1	P00352
aldehyde dehydrogenase 2 family member	ALDH2	P05091
aldehyde dehydrogenase 7 family member A1	ALDH7A1	P49419
aldehyde dehydrogenase 9 family member A1	ALDH9A1	P49189
aldo-keto reductase family 1 member B	AKR1B1	P15121
aldo-keto reductase family 1 member C2	AKR1C2	P52895
Aldo-keto reductase family 1 member C2	AKR1C2	P52895
aldo-keto reductase family 1 member C3	AKR1C3	P42330
Aldo-keto reductase family 1 member C3	AKR1C3	P42330
aldolase, fructose-bisphosphate A	ALDOA	P04075
Aldose reductase	AKR1B1	P15121

Target name	Gene Name	UniProt ID
alkaline phosphatase, biomineralization associated	ALPL	P05186
alkaline phosphatase, germ cell	ALPG	P10696
alkaline phosphatase, intestinal	ALPI	P09923
alkaline phosphatase, placental	ALPP	P05187
Amine oxidase [flavin-containing] B	MAOB	P27338
aminolevulinate dehydratase	ALAD	P13716
Amyloid-beta precursor protein	APP	P05067
androgen receptor	AR	P10275
angiopoietin 1	ANGPT1	Q15389
angiopoietin 2	ANGPT2	O15123
ankyrin 1	ANK1	P16157
ankyrin 2	ANK2	Q01484
ankyrin 3	ANK3	Q12955
ankyrin repeat and BTB domain containing 1	ABTB1	Q969K4
ankyrin repeat and death domain containing 1B	ANKDD1B	A6NHY2
ankyrin repeat and EF-hand domain containing 1	ANKEF1	Q9NU02
ankyrin repeat and FYVE domain containing 1	ANKFY1	Q9P2R3
ankyrin repeat and KH domain containing 1	ANKHD1	Q8IWZ3
ankyrin repeat and kinase domain containing 1	ANKK1	Q8NFD2
ankyrin repeat and SOCS box containing 1	ASB1	Q9Y576
ankyrin repeat and SOCS box containing 10	ASB10	Q8WXI3
ankyrin repeat and SOCS box containing 11	ASB11	Q8WXH4
ankyrin repeat and SOCS box containing 12	ASB12	Q8WXK4
ankyrin repeat and SOCS box containing 13	ASB13	Q8WXK3
ankyrin repeat and SOCS box containing 14	ASB14	A6NK59

Target name	Gene Name	UniProt ID
ankyrin repeat and SOCS box containing 15	ASB15	Q8WXK1
ankyrin repeat and SOCS box containing 16	ASB16	Q96NS5
ankyrin repeat and SOCS box containing 18	ASB18	Q6ZVZ8
ankyrin repeat and SOCS box containing 2	ASB2	Q96Q27
ankyrin repeat and SOCS box containing 4	ASB4	Q9Y574
ankyrin repeat and SOCS box containing 5	ASB5	Q8WWX0
ankyrin repeat and SOCS box containing 6	ASB6	Q9NWX5
ankyrin repeat and SOCS box containing 7	ASB7	Q9H672
ankyrin repeat and SOCS box containing 8	ASB8	Q9H765
ankyrin repeat and SOCS box containing 9	ASB9	Q96DX5
ankyrin repeat and sterile alpha motif domain containing 1A	ANKS1A	Q92625
ankyrin repeat and sterile alpha motif domain containing 1B	ANKS1B	Q7Z6G8
ankyrin repeat and sterile alpha motif domain containing 3	ANKS3	Q6ZW76
ankyrin repeat and sterile alpha motif domain containing 4B	ANKS4B	Q8N8V4
ankyrin repeat and sterile alpha motif domain containing 6	ANKS6	Q68DC2
ankyrin repeat domain 1	ANKRD1	Q15327
ankyrin repeat domain 10	ANKRD10	Q9NXR5
ankyrin repeat domain 16	ANKRD16	Q6P6B7
ankyrin repeat domain 17	ANKRD17	O75179
ankyrin repeat domain 18A	ANKRD18A	Q8IVF6
ankyrin repeat domain 18B	ANKRD18B	A2A2Z9
ankyrin repeat domain 2	ANKRD2	Q9GZV1
ankyrin repeat domain 20 family member A2, pseudogene	ANKRD20A2P	Q5SQ80
ankyrin repeat domain 20 family member A4, pseudogene	ANKRD20A4P	Q4UJ75
ankyrin repeat domain 22	ANKRD22	Q5VYY1

Target name	Gene Name	UniProt ID
ankyrin repeat domain 23	ANKRD23	Q86SG2
ankyrin repeat domain 24	ANKRD24	Q8TF21
ankyrin repeat domain 26	ANKRD26	Q9UPS8
ankyrin repeat domain 27	ANKRD27	Q96NW4
ankyrin repeat domain 28	ANKRD28	O15084
ankyrin repeat domain 29	ANKRD29	Q8N6D5
ankyrin repeat domain 30A	ANKRD30A	Q9BXX3
ankyrin repeat domain 30B	ANKRD30B	Q9BXX2
ankyrin repeat domain 30B like	ANKRD30BL	A7E2S9
ankyrin repeat domain 35	ANKRD35	Q8N283
ankyrin repeat domain 36	ANKRD36	A6QL64
ankyrin repeat domain 36C	ANKRD36C	Q5JPF3
ankyrin repeat domain 37	ANKRD37	Q7Z713
ankyrin repeat domain 39	ANKRD39	Q53RE8
ankyrin repeat domain 42	ANKRD42	Q8N9B4
ankyrin repeat domain 44	ANKRD44	Q8N8A2
ankyrin repeat domain 46	ANKRD46	Q86W74
ankyrin repeat domain 49	ANKRD49	Q8WVL7
ankyrin repeat domain 50	ANKRD50	Q9ULJ7
ankyrin repeat domain 52	ANKRD52	Q8NB46
ankyrin repeat domain 53	ANKRD53	Q8N9V6
ankyrin repeat domain 54	ANKRD54	Q6NXT1
ankyrin repeat domain 55	ANKRD55	Q3KP44
ankyrin repeat domain 6	ANKRD6	Q9Y2G4
ankyrin repeat domain 60	ANKRD60	Q9BZ19

Target name	Gene Name	UniProt ID
ankyrin repeat domain 61	ANKRD61	A6NGH8
ankyrin repeat domain 63	ANKRD63	C9JTQ0
ankyrin repeat domain 65	ANKRD65	E5RJM6
ankyrin repeat domain 66	ANKRD66	B4E2M5
ankyrin repeat domain 7	ANKRD7	Q92527
ankyrin repeat domain 9	ANKRD9	Q96BM1
ankyrin repeat family A member 2	ANKRA2	Q9H9E1
ankyrin repeat, SAM and basic leucine zipper domain containing 1	ASZ1	Q8WWH4
annexin A3	ANXA3	P12429
annexin A5	ANXA5	P08758
APAF1 interacting protein	APIP	Q96GX9
apoptosis inducing factor mitochondria associated 1	AIFM1	O95831
Apoptosis regulator BAX	BAX	Q07812
Apoptosis regulator Bcl-2	BCL2	P10415
apoptotic peptidase activating factor 1	APAF1	O14727
Apoptotic protease-activating factor 1	APAF1	O14727
arachidonate 5-lipoxygenase	ALOX5	P09917
Arachidonate 5-lipoxygenase	ALOX5AP	P20292
arginase 1	ARG1	P05089
Aromatase	CYP19A1	P11511
aryl hydrocarbon receptor	AHR	P35869
asparaginase	ASPG	Q86U10
asparaginase and isoaspartyl peptidase 1	ASRGL1	Q7L266
Aspartyl aminopeptidase	DNPEP	Q9ULA0
ATM interactor	ATMIN	O43313

Target name	Gene Name	UniProt ID
ATM serine/threonine kinase	ATM	Q13315
ATP binding cassette subfamily B member 1	ABCB1	P08183
ATP synthase membrane subunit c locus 2	ATP5MC2	Q06055
ATP/GTP binding protein 1	AGTPBP1	Q9UPW5
ATP/GTP binding protein like 1	AGBL1	Q96MI9
ATP/GTP binding protein like 3	AGBL3	Q8NEM8
ATP/GTP binding protein like 4	AGBL4	Q5VU57
ATP/GTP binding protein like 5	AGBL5	Q8NDL9
ATP-binding cassette sub-family G member 2	Abcg2	G0TEB1
ATR serine/threonine kinase	ATR	Q13535
autophagy related 5	ATG5	Q9H1Y0
AXL receptor tyrosine kinase	AXL	P30530
azurocidin 1	AZU1	P20160
baculoviral IAP repeat containing 2	BIRC2	Q13490
baculoviral IAP repeat containing 5	BIRC5	O15392
baculoviral IAP repeat containing 8	BIRC8	#N/A
Baculoviral IAP repeat-containing protein 5	BIRC5	O15392
BAG cochaperone 3	BAG3	O95817
BCL2 antagonist/killer 1	BAK1	Q16611
BCL2 apoptosis regulator	BCL2	P10415
BCL2 associated agonist of cell death	BAD	Q92934
BCL2 associated transcription factor 1	BCLAF1	Q9NYF8
BCL2 associated X, apoptosis regulator	BAX	Q07812
BCL2 interacting protein 2	BNIP2	Q12982
BCL2 interacting protein 3	BNIP3	Q12983

Target name	Gene Name	UniProt ID
BCL2 interacting protein 3 like	BNIP3L	O60238
BCL2 like 1	BCL2L1	Q07817
Bcl-2-like protein 1	BCL2L1	Q07817
BCL3 transcription coactivator	BCL3	P20749
BDNF/NT-3 growth factors receptor	NTRK2	Q16620
beclin 1	BECN1	Q14457
Beta-1 adrenergic receptor	ADRB1	P08588
beta-1,3-galactosyltransferase 4	B3GALT4	O96024
Beta-2 adrenergic receptor	ADRB2	P07550
betaine--homocysteine S-methyltransferase	BHMT	Q93088
betaine--homocysteine S-methyltransferase 2	BHMT2	Q9H2M3
Beta-secretase 1	BACE1	P56817
BH3 interacting domain death agonist	BID	P55957
Bile acid receptor	NR1H4	Q96RI1
BLK proto-oncogene, Src family tyrosine kinase	BLK	P51451
BMX non-receptor tyrosine kinase	BMX	P51813
bradykinin receptor B1	BDKRB1	P46663
Brain-derived neurotrophic factor	BDNF	P23560
BRCA1 associated RING domain 1	BARD1	Q99728
Bruton tyrosine kinase	BTK	Q06187
BTG3 associated nuclear protein	BANP	Q8N9N5
butyrylcholinesterase	BCHE	P06276
C1q and TNF related 5	C1QTNF5	Q9BXJ0
calcitonin related polypeptide alpha	CALCA	P06881
calcitonin related polypeptide beta	CALCB	P10092

**Target name**	**Gene Name**	**UniProt ID**
calcium voltage-gated channel subunit alpha1 H	CACNA1H	O95180
Calmodulin	CALM1	P0DP23
calmodulin 3	CALM3	P0DP25
cAMP responsive element binding protein 1	CREB1	P16220
cAMP-dependent protein kinase inhibitor alpha	PKIA	P61925
Cannabinoid receptor 1	CNR1	P21554
Cannabinoid receptor 2	CNR2	P34972
Carbonic anhydrase 1	CA1	P00915
Carbonic anhydrase 12	CA12	O43570
carbonic anhydrase 2	CA2	P00918
Carbonic anhydrase 4	CA4	P22748
Carbonic anhydrase 7	CA7	P43166
Carbonic anhydrase 9	CA9	Q16790
carboxylesterase 2	CES2	O00748
carboxypeptidase A1	CPA1	P15085
carboxypeptidase A2	CPA2	P48052
carboxypeptidase A3	CPA3	P15088
carboxypeptidase A4	CPA4	Q9UI42
carboxypeptidase A6	CPA6	Q8N4T0
carboxypeptidase B1	CPB1	P15086
carboxypeptidase B2	CPB2	Q96IY4
carboxypeptidase O	CPO	Q8IVL8
carnitine palmitoyltransferase 1A	CPT1A	P50416
CASK interacting protein 1	CASKIN1	Q8WXD9
CASK interacting protein 2	CASKIN2	Q8WXE0

Target name	Gene Name	UniProt ID
CASP8 and FADD like apoptosis regulator	CFLAR	O15519
caspase 12 (gene/pseudogene)	CASP12	#N/A
caspase 3	CASP3	P42574
caspase 4	CASP4	P49662
caspase 7	CASP7	P55210
caspase 8	CASP8	Q14790
caspase 9	CASP9	P55211
catalase	CAT	P04040
catenin beta 1	CTNNB1	P35222
cathepsin B	CTSB	P07858
cathepsin D	CTSD	P07339
cathepsin G	CTSG	P08311
Caveolin-1	CAV1	Q03135
Cbp/p300-interacting transactivator 1	CITED1	Q99966
C-C motif chemokine 2	CCL2	P13500
CD40 ligand	CD40LG	P29965
CDC42 binding protein kinase beta	CDC42BPB	Q9Y5S2
Cell division control protein 2 homolog	CDC2	P93101
cell division cycle 25A	CDC25A	P30304
cell division cycle 25B	CDC25B	P30305
cell division cycle 42	CDC42	P60953
cell migration inducing hyaluronidase 1	CEMIP	Q8WUJ3
Cell-death-related nuclease 7	CASP7	P55210
Cellular tumor antigen p53	TP53	P04637
CGMP-inhibited 3',5'-cyclic phosphodiesterase A	PDE3A	Q14432

Target name	Gene Name	UniProt ID
cGMP-specific 3',5'-cyclic phosphodiesterase	PDE5A	O76074
checkpoint kinase 1	CHEK1	O14757
checkpoint kinase 2	CHEK2	O96017
cholecystokinin	CCK	P06307
Cholesteryl ester transfer protein	CETP	P11597
cholinergic receptor muscarinic 1	CHRM1	P11229
cholinergic receptor muscarinic 2	CHRM2	P08172
cholinergic receptor muscarinic 3	CHRM3	P20309
cholinergic receptor muscarinic 4	CHRM4	P08173
cholinergic receptor muscarinic 5	CHRM5	P08912
cholinergic receptor nicotinic alpha 2 subunit	CHRNA2	Q15822
cholinergic receptor nicotinic alpha 7 subunit	CHRNA7	P36544
chymase 1	CMA1	P23946
chymotrypsin C	CTRC	Q99895
chymotrypsin like	CTRL	P40313
chymotrypsin like elastase 1	CELA1	Q9UNI1
chymotrypsin like elastase 2A	CELA2A	P08217
chymotrypsin like elastase 2B	CELA2B	P08218
chymotrypsin like elastase 3A	CELA3A	P09093
chymotrypsin like elastase 3B	CELA3B	P08861
chymotrypsinogen B1	CTRB1	P17538
chymotrypsinogen B2	CTRB2	Q6GPI1
Claudin-4	CLDN4	O14493
coagulation factor II, thrombin	F2	P00734
coagulation factor IX	F9	P00740

Target name	Gene Name	UniProt ID
coagulation factor VII	F7	P08709
coagulation factor X	F10	P00742
Coagulation factor X	F10	P00742
Coagulation factor Xa	F10	P00742
coagulation factor XI	F11	P03951
coagulation factor XII	F12	P00748
coagulation factor XIII A chain	F13A1	P00488
Cocaine esterase	CES2	O00748
cofilin 1	CFL1	P23528
Collagen alpha-1(I) chain	COL1A1	P02452
Collagen alpha-1(III) chain	COL3A1	P02461
collagen type XVIII alpha 1 chain	COL18A1	P39060
colony stimulating factor 1 receptor	CSF1R	P07333
colony stimulating factor 2	CSF2	P04141
complement C1r subcomponent like	C1RL	Q9NZP8
complement C1s	C1S	P09871
complement C2	C2	P06681
complement factor B	CFB	P00751
complement factor D	CFD	P00746
complement factor I	CFI	P05156
component of inhibitor of nuclear factor kappa B kinase complex	CHUK	Q60680
corin, serine peptidase	CORIN	Q9Y5Q5
cortactin binding protein 2	CTTNBP2	Q8WZ74
C-reactive protein	CRP	P02741
creatine kinase B	CKB	P12277

Target name	Gene Name	UniProt ID
creatine kinase, mitochondrial 1A	CKMT1A	ENSG00000223572
creatine kinase, mitochondrial 2	CKMT2	P17540
creatine kinase, M-type	CKM	P06732
C-terminal Src kinase	CSK	P41240
C-X-C motif chemokine 10	CXCL10	P02778
C-X-C motif chemokine 11	CXCL11	O14625
C-X-C motif chemokine 2	CXCL2	P19875
C-X-C motif chemokine ligand 8	CXCL8	P10145
cyclin A1	CCNA1	P78396
cyclin A2	CCNA2	P20248
cyclin B1	CCNB1	P14635
cyclin D1	CCND1	P24385
cyclin D2	CCND2	P30279
cyclin dependent kinase 1	CDK1	P06493
cyclin dependent kinase 2	CDK2	P24941
cyclin dependent kinase 4	CDK4	P11802
cyclin dependent kinase 6	CDK6	Q00534
cyclin dependent kinase inhibitor 1A	CDKN1A	P38936
cyclin dependent kinase inhibitor 1B	CDKN1B	P46527
cyclin dependent kinase inhibitor 2B	CDKN2B	P42772
cyclin dependent kinase inhibitor 2C	CDKN2C	P42773
cyclin dependent kinase inhibitor 2D	CDKN2D	P55273
cyclin E1	CCNE1	P24864
Cyclin-dependent kinase inhibitor 1	CDKN1A	P38936
Cyclin-dependent kinase inhibitor 2A, isoforms 1/2/3	CDKN2A	P42771

Target name	Gene Name	UniProt ID
cystathionine beta-synthase	CBS	P35520
cystathionine gamma-lyase	CTH	P32929
cytochrome c oxidase subunit 5A	COX5A	P20674
cytochrome c, somatic	CYCS	P99999
Cytochrome P450 1A1	CYP1A1	P04798
Cytochrome P450 1A1	CYP1A1	P04798
Cytochrome P450 1A2	CYP1A2	P05177
Cytochrome P450 1B1	CYP1B1	Q16678
Cytochrome P450 1B1	CYP1B1	Q16678
Cytochrome P450 2C9	CYP2C9	P11712
Cytochrome P450 3A4	CYP3A4	P08684
Cytochrome P450 3A4	CYP3A4	P08684
cytochrome P450 family 1 subfamily A member 1	CYP1A1	P04798
cytochrome P450 family 1 subfamily A member 2	CYP1A2	P05177
cytochrome P450 family 11 subfamily A member 1	CYP11A1	P05108
cytochrome P450 family 11 subfamily B member 1	CYP11B1	P15538
cytochrome P450 family 11 subfamily B member 2	CYP11B2	P19099
cytochrome P450 family 17 subfamily A member 1	CYP17A1	P05093
cytochrome P450 family 2 subfamily A member 13	CYP2A13	Q16696
cytochrome P450 family 2 subfamily A member 6	CYP2A6	P11509
cytochrome P450 family 2 subfamily A member 7	CYP2A7	P20853
cytochrome P450 family 2 subfamily B member 6	CYP2B6	P20813
cytochrome P450 family 2 subfamily C member 18	CYP2C18	P33260
cytochrome P450 family 2 subfamily C member 19	CYP2C19	P33261
cytochrome P450 family 2 subfamily C member 8	CYP2C8	P10632

Target name	Gene Name	UniProt ID
cytochrome P450 family 2 subfamily C member 9	CYP2C9	P11712
cytochrome P450 family 2 subfamily D member 6	CYP2D6	P10635
cytochrome P450 family 2 subfamily E member 1	CYP2E1	P05181
cytochrome P450 family 2 subfamily F member 1	CYP2F1	P24903
cytochrome P450 family 2 subfamily J member 2	CYP2J2	P51589
cytochrome P450 family 2 subfamily R member 1	CYP2R1	Q6VVX0
cytochrome P450 family 2 subfamily S member 1	CYP2S1	Q96SQ9
cytochrome P450 family 2 subfamily U member 1	CYP2U1	Q7Z449
cytochrome P450 family 2 subfamily W member 1	CYP2W1	Q8TAV3
cytochrome P450 family 20 subfamily A member 1	CYP20A1	Q6UW02
cytochrome P450 family 24 subfamily A member 1	CYP24A1	Q07973
cytochrome P450 family 26 subfamily A member 1	CYP26A1	O43174
cytochrome P450 family 26 subfamily B member 1	CYP26B1	Q9NR63
cytochrome P450 family 26 subfamily C member 1	CYP26C1	Q6V0L0
cytochrome P450 family 27 subfamily A member 1	CYP27A1	Q02318
cytochrome P450 family 27 subfamily B member 1	CYP27B1	O15528
cytochrome P450 family 27 subfamily C member 1	CYP27C1	Q4G0S4
cytochrome P450 family 3 subfamily A member 4	CYP3A4	P08684
cytochrome P450 family 3 subfamily A member 43	CYP3A43	Q9HB55
cytochrome P450 family 3 subfamily A member 5	CYP3A5	P20815
cytochrome P450 family 3 subfamily A member 7	CYP3A7	P24462
cytochrome P450 family 39 subfamily A member 1	CYP39A1	Q9NYL5
cytochrome P450 family 4 subfamily A member 11	CYP4A11	Q02928
cytochrome P450 family 4 subfamily A member 22	CYP4A22	Q5TCH4
cytochrome P450 family 4 subfamily B member 1	CYP4B1	P13584

Target name	Gene Name	UniProt ID
cytochrome P450 family 4 subfamily F member 11	CYP4F11	Q9HBI6
cytochrome P450 family 4 subfamily F member 12	CYP4F12	Q9HCS2
cytochrome P450 family 4 subfamily F member 2	CYP4F2	P78329
cytochrome P450 family 4 subfamily F member 22	CYP4F22	Q6NT55
cytochrome P450 family 4 subfamily F member 3	CYP4F3	Q08477
cytochrome P450 family 4 subfamily V member 2	CYP4V2	Q6ZWL3
cytochrome P450 family 4 subfamily X member 1	CYP4X1	Q8N118
cytochrome P450 family 4 subfamily Z member 1	CYP4Z1	Q86W10
cytochrome P450 family 46 subfamily A member 1	CYP46A1	Q9Y6A2
cytochrome P450 family 51 subfamily A member 1	CYP51A1	Q16850
cytochrome P450 family 7 subfamily A member 1	CYP7A1	P22680
cytochrome P450 family 7 subfamily B member 1	CYP7B1	O75881
cytochrome P450 family 8 subfamily B member 1	CYP8B1	Q9UNU6
cytochrome p450 oxidoreductase	POR	P16435
Cytochrome P450-cam	NOR51B_261	B8KSV6
D(4) dopamine receptor	DRD4	P21917
D-amino acid oxidase	DAO	P14920
DDB1- and CUL4-associated factor 5	DCAF5	Q96JK2
death domain associated protein	DAXX	Q9UER7
dipeptidase 1	DPEP1	P16444
dipeptidyl peptidase 4	DPP4	P27487
Dipeptidyl peptidase IV	DPP4	P27487
DNA damage inducible transcript 3	DDIT3	P35638
DNA dC->dU-editing enzyme APOBEC-3A	APOBEC3A	P31941
DNA fragmentation factor subunit alpha	DFFA	O00273

Target name	Gene Name	UniProt ID
DNA gyrase subunit B	gyrB	P0AES6
DNA ligase 1	LIG1	P18858
DNA polymerase alpha catalytic subunit	POLA1	P09884
DNA topoisomerase 1	TOP1	P11387
DNA topoisomerase 2-alpha	TOP2A	P11388
DNA topoisomerase I	TOP1	P11387
Dopamine D1 receptor	DRD1	P21728
dopamine receptor D1	DRD1	P21728
Dual oxidase 2	DUOX2	Q9NRD8
dual serine/threonine and tyrosine protein kinase	DSTYK	Q6XUX3
dynamin 1 like	DNM1L	O00429
E1A binding protein p300	EP300	Q09472
E2F transcription factor 1	E2F1	Q01094
E2F transcription factor 4	E2F4	Q16254
ecto-NOX disulfide-thiol exchanger 2	ENOX2	Q16206
elastase, neutrophil expressed	ELANE	P08246
endoplasmic reticulum to nucleus signaling 1	ERN1	O75460
endoplasmic reticulum to nucleus signaling 2	ERN2	Q76MJ5
endothelin 1	EDN1	P05305
Endothelin-1	EDN1	P05305
EPH receptor A1	EPHA1	P21709
EPH receptor A10	EPHA10	Q5JZY3
EPH receptor A2	EPHA2	P29317
EPH receptor A3	EPHA3	P29320
EPH receptor A4	EPHA4	P54764

Target name	Gene Name	UniProt ID
EPH receptor A5	EPHA5	P54756
EPH receptor A6	EPHA6	Q9UF33
EPH receptor A7	EPHA7	Q15375
EPH receptor A8	EPHA8	P29322
EPH receptor B1	EPHB1	P54762
EPH receptor B2	EPHB2	P29323
EPH receptor B3	EPHB3	P54753
EPH receptor B4	EPHB4	P54760
EPH receptor B6	EPHB6	ENSG00000275482
epidermal growth factor receptor	EGFR	P00533
Epidermal growth factor receptor	EGFR	P00533
epoxide hydrolase 1	EPHX1	P07099
erb-b2 receptor tyrosine kinase 2	ERBB2	P04626
erythropoietin receptor	EPOR	P19235
E-selectin	SELE	P16581
espin	ESPN	B1AK53
espin like	ESPNL	Q6ZVH7
Estrogen receptor	ESR1	P03372
estrogen receptor 1	ESR1	P03372
estrogen receptor 2	ESR2	Q92731
Estrogen sulfotransferase	SULT1E1	P49888
ETS domain-containing protein Elk-1	ELK1	P19419
euchromatic histone lysine methyltransferase 1	EHMT1	Q9H9B1
euchromatic histone lysine methyltransferase 2	EHMT2	Q96KQ7
eukaryotic translation initiation factor 2 alpha kinase 3	EIF2AK3	Q9NZJ5

Target name	Gene Name	UniProt ID
eukaryotic translation initiation factor 4E binding protein 1	EIF4EBP1	Q13541
Eukaryotic translation initiation factor 6	EIF6	P56537
F2R like trypsin receptor 1	F2RL1	P55085
Fas cell surface death receptor	FAS	P25445
Fas ligand	FASLG	P48023
fatty acid binding protein 4	FABP4	P15090
fatty acid binding protein 5	FABP5	Q01469
fatty acid synthase	FASN	P49327
Fatty acid-binding protein, liver	FABP1	P07148
fem-1 homolog A	FEM1A	Q9BSK4
fem-1 homolog B	FEM1B	Q9UK73
fem-1 homolog C	FEM1C	Q96JP0
ferredoxin reductase	FDXR	P22570
FGR proto-oncogene, Src family tyrosine kinase	FGR	P09769
fibroblast activation protein alpha	FAP	Q12884
fibroblast growth factor receptor 1	FGFR1	P11362
fibroblast growth factor receptor 2	FGFR2	P21802
fibroblast growth factor receptor 3	FGFR3	P22607
fibroblast growth factor receptor 4	FGFR4	P22455
fibroblast growth factor receptor like 1	FGFRL1	Q8N441
fibronectin 1	FN1	P02751
fibronectin type III and ankyrin repeat domains 1	FANK1	Q8TC84
FKBP prolyl isomerase 1A	FKBP1A	P62942
fms related receptor tyrosine kinase 1	FLT1	P17948
fms related receptor tyrosine kinase 3	FLT3	P36888

Target name	Gene Name	UniProt ID
fms related receptor tyrosine kinase 4	FLT4	P35916
forkhead box A1	FOXA1	P55317
forkhead box P3	FOXP3	Q9BZS1
Fos proto-oncogene, AP-1 transcription factor subunit	FOS	P01100
FPGT-TNNI3K readthrough	FPGT-TNNI3K	ENSG00000259030
free fatty acid receptor 4	FFAR4	Q5NUL3
frizzled class receptor 1	FZD1	Q9UP38
frizzled class receptor 10	FZD10	Q9ULW2
frizzled class receptor 2	FZD2	Q14332
frizzled class receptor 3	FZD3	Q9NPG1
frizzled class receptor 4	FZD4	Q9ULV1
frizzled class receptor 5	FZD5	Q13467
frizzled class receptor 6	FZD6	O60353
frizzled class receptor 7	FZD7	O75084
frizzled class receptor 8	FZD8	Q9H461
frizzled class receptor 9	FZD9	O00144
frizzled related protein	FRZB	Q92765
furin, paired basic amino acid cleaving enzyme	FURIN	P09958
FYN proto-oncogene, Src family tyrosine kinase	FYN	P06241
GA binding protein transcription factor subunit alpha	GABPA	Q06546
GA binding protein transcription factor subunit beta 1	GABPB1	Q06547
GA binding protein transcription factor subunit beta 2	GABPB2	Q8TAK5
galactosidase beta 1	GLB1	P16278
galanin and GMAP prepropeptide	GAL	P22466
galectin 1	LGALS1	P09382

Target name	Gene Name	UniProt ID
Gamma-aminobutyric acid receptor subunit alpha-1	GABRA1	P14867
gamma-aminobutyric acid type A receptor subunit alpha1	GABRA1	P14867
Gamma-aminobutyric-acid receptor alpha-2 subunit	GABRA2	P47869
Gamma-aminobutyric-acid receptor alpha-3 subunit	GABRA3	P34903
Gamma-aminobutyric-acid receptor alpha-5 subunit	GABRA5	P31644
Gap junction alpha-1 protein	GJA1	P17302
gastrin	GAST	P01350
GATA binding protein 1	GATA1	P15976
GATA binding protein 2	GATA2	P23769
GATA binding protein 3	GATA3	P23771
gephyrin	GPHN	Q9NQX3
glial fibrillary acidic protein	GFAP	P14136
glucagon	GCG	P01275
glucosamine-6-phosphate deaminase 1	GNPDA1	P46926
glucose-6-phosphate isomerase	GPI	P06744
glutamate dehydrogenase 1	GLUD1	P00367
glutamate dehydrogenase 2	GLUD2	P49448
glutamate ionotropic receptor AMPA type subunit 2	GRIA2	P42262
glutamate ionotropic receptor NMDA type subunit 2A	GRIN2A	Q12879
glutamate ionotropic receptor NMDA type subunit 2C	GRIN2C	Q14957
glutamate ionotropic receptor NMDA type subunit 2D	GRIN2D	O15399
glutamate ionotropic receptor NMDA type subunit 3A	GRIN3A	Q8TCU5
glutamate ionotropic receptor NMDA type subunit 3B	GRIN3B	O60391
Glutamate receptor ionotropic, NMDA 1	GRIN1	Q05586
glutamate-ammonia ligase	GLUL	P15104

Target name	Gene Name	UniProt ID
glutamate-cysteine ligase modifier subunit	GCLM	P48507
glutamic-oxaloacetic transaminase 2	GOT2	P00505
glutamic--pyruvic transaminase	GPT	P24298
glutamic--pyruvic transaminase 2	GPT2	Q8TD30
Glutamyl aminopeptidase	Enpep	P16406
glutathione S-transferase alpha 1	GSTA1	P08263
glutathione S-transferase alpha 2	GSTA2	P09210
glutathione S-transferase mu 1	GSTM1	P09488
Glutathione S-transferase Mu 2	GSTM2	P28161
Glutathione S-transferase P	GSTP1	P09211
glutathione S-transferase pi 1	GSTP1	P09211
glutathione S-transferase zeta 1	GSTZ1	O43708
glutathione-disulfide reductase	GSR	P00390
glyceraldehyde-3-phosphate dehydrogenase	GAPDH	P04406
glycine amidinotransferase	GATM	P50440
glycine C-acetyltransferase	GCAT	O75600
glycine decarboxylase	GLDC	P23378
glycine N-methyltransferase	GNMT	Q14749
glycine receptor alpha 2	GLRA2	P23416
glycine receptor alpha 3	GLRA3	O75311
glycogen synthase kinase 3 alpha	GSK3A	P49840
glycogen synthase kinase 3 beta	GSK3B	P49841
Glycogen synthase kinase-3 beta	GSK3B	P49841
glycophorin A (MNS blood group)	GYPA	P02724
GM2 ganglioside activator	GM2A	P17900

Target name	Gene Name	UniProt ID
G-protein coupled bile acid receptor 1	GPBAR1	Q8TDU6
G-protein coupled receptor 55	GPR55	Q9Y2T6
granzyme A	GZMA	P12544
granzyme B	GZMB	P10144
granzyme H	GZMH	P20718
granzyme K	GZMK	P49863
granzyme M	GZMM	P51124
growth arrest and DNA damage inducible alpha	GADD45A	P24522
growth associated protein 43	GAP43	P17677
growth differentiation factor 15	GDF15	Q99988
growth factor receptor bound protein 2	GRB2	P62993
guanidinoacetate N-methyltransferase	GAMT	Q14353
H2A.X variant histone	H2AX	P16104
haptoglobin	HP	P00738
haptoglobin-related protein	HPR	P00739
HCK proto-oncogene, Src family tyrosine kinase	HCK	P08631
Heat shock factor protein 1	HSF1	Q00613
heat shock protein 90 beta family member 1	HSP90B1	P14625
Heat shock protein beta-1	HSPB1	P04792
heat shock protein family A (Hsp70) member 5	HSPA5	P11021
Heat shock protein HSP 90	HSP90AA1	P07900
Heat shock protein HSP 90-alpha	HSP90AA1	P07900
HECT domain and ankyrin repeat containing E3 ubiquitin protein ligase 1	HACE1	Q8IYU2
heme oxygenase 1	HMOX1	P09601
hemoglobin subunit beta	HBB	P68871

Target name	Gene Name	UniProt ID
hepatocyte growth factor	HGF	P14210
hepatocyte nuclear factor 4 alpha	HNF4A	P41235
hepatocyte nuclear factor 4 gamma	HNF4G	Q14541
hepsin	HPN	P05981
Hexokinase-2	HK2	P52789
hexose-6-phosphate dehydrogenase/glucose 1-dehydrogenase	H6PD	O95479
HGF activator	HGFAC	Q04756
Histamine H2 receptor	HRH2	P25021
Homeobox protein Nkx-3.1	NKX3-1	Q99801
hormonally up-regulated Neu-associated kinase	HUNK	P57058
HRas proto-oncogene, GTPase	HRAS	P01112
hyaluronan binding protein 2	HABP2	Q14520
Hyaluronan synthase 2	HAS2	Q92819
hydroxyacid oxidase 1	HAO1	Q9UJM8
hydroxyacyl-CoA dehydrogenase trifunctional multienzyme complex subunit beta	HADHB	P55084
hydroxy-delta-5-steroid dehydrogenase, 3 beta- and steroid delta-isomerase 2	HSD3B2	P26439
hypoxia inducible factor 1 subunit alpha	HIF1A	Q16665
Hypoxia-inducible factor 1-alpha	HIF1A	Q16665
IL2 inducible T cell kinase	ITK	Q08881
immunoglobulin heavy constant gamma 1 (G1m marker)	IGHG1	#N/A
Induced myeloid leukemia cell differentiation protein Mcl-1	MCL1	Q07820
Inhibitor of nuclear factor kappa-B kinase subunit alpha	Chuk	Q60680
inositol-3-phosphate synthase 1	ISYNA1	Q9NPH2
insulin	INS	P01308
insulin like growth factor 1	IGF1	P05019

Target name	Gene Name	UniProt ID
insulin like growth factor 1 receptor	IGF1R	P08069
insulin like growth factor binding protein 1	IGFBP1	P08833
insulin receptor	INSR	P06213
insulin receptor related receptor	INSRR	P14616
Insulin-like growth factor II	IGF2	P01344
Insulin-like growth factor-binding protein 3	IGFBP3	P17936
Integrin beta-3	ITGB3	P05106
integrin linked kinase	ILK	Q13418
integrin subunit alpha 5	ITGA5	P08648
integrin subunit alpha M	ITGAM	P11215
Intercellular adhesion molecule 1	ICAM1	P05362
Interferon gamma	IFNG	P01579
Interferon regulatory factor 1	IRF1	P10914
interleukin 1 receptor associated kinase 1	IRAK1	P51617
interleukin 1 receptor associated kinase 2	IRAK2	O43187
interleukin 1 receptor associated kinase 3	IRAK3	Q9Y616
interleukin 1 receptor associated kinase 4	IRAK4	Q9NWZ3
interleukin 12B	IL12B	P29460
interleukin 17A	IL17A	Q16552
interleukin 18	IL18	Q14116
interleukin 2	IL2	P60568
interleukin 6	IL6	P05231
Interleukin-1 alpha	IL1A	P01583
Interleukin-1 beta	IL1B	P01584
Interleukin-10	IL10	P22301

Target name	Gene Name	UniProt ID
Interleukin-2	IL2	P60568
Interleukin-6	IL6	P05231
Interleukin-8	CXCL8	P10145
Intermediate conductance calcium-activated potassium channel protein 4	KCNN4	O15554
Interstitial collagenase	MMP1	P03956
inversin	INVS	Q9Y283
Janus kinase 1	JAK1	P23458
Janus kinase 2	JAK2	O60674
Janus kinase 3	JAK3	P52333
Jun proto-oncogene, AP-1 transcription factor subunit	JUN	P05412
JunB proto-oncogene, AP-1 transcription factor subunit	JUNB	P17275
kallikrein 1	KLK1	P06870
kallikrein B1	KLKB1	P03952
kallikrein related peptidase 10	KLK10	O43240
kallikrein related peptidase 11	KLK11	Q9UBX7
kallikrein related peptidase 12	KLK12	Q9UKR0
kallikrein related peptidase 13	KLK13	Q9UKR3
kallikrein related peptidase 14	KLK14	Q9P0G3
kallikrein related peptidase 15	KLK15	Q9H2R5
kallikrein related peptidase 2	KLK2	P20151
kallikrein related peptidase 3	KLK3	P07288
kallikrein related peptidase 4	KLK4	Q9Y5K2
kallikrein related peptidase 5	KLK5	Q9Y337
kallikrein related peptidase 6	KLK6	Q92876
kallikrein related peptidase 7	KLK7	P49862

Target name	Gene Name	UniProt ID
kallikrein related peptidase 8	KLK8	O60259
kallikrein related peptidase 9	KLK9	Q9UKQ9
Kappa-type opioid receptor	OPRK1	P41145
kinase D interacting substrate 220	KIDINS220	Q9ULH0
kinase insert domain receptor	KDR	P35968
KIT proto-oncogene, receptor tyrosine kinase	KIT	P10721
KN motif and ankyrin repeat domains 1	KANK1	Q14678
KN motif and ankyrin repeat domains 3	KANK3	Q6NY19
KN motif and ankyrin repeat domains 4	KANK4	Q5T7N3
kynureninase	KYNU	Q16719
kynurenine aminotransferase 1	KYAT1	Q16773
lactate dehydrogenase A	LDHA	P00338
lactate dehydrogenase B	LDHB	P07195
lactotransferrin	LTF	P02788
LCK proto-oncogene, Src family tyrosine kinase	LCK	P06239
leucine aminopeptidase 3	LAP3	P28838
leucine carboxyl methyltransferase 2	LCMT2	O60294
leukotriene A4 hydrolase	LTA4H	P09960
leukotriene C4 synthase	LTC4S	Q16873
LIM domain kinase 1	LIMK1	P53667
LIM domain kinase 2	LIMK2	P53671
lipoprotein lipase	LPL	P06858
lipoprotein(a)	LPA	P08519
low density lipoprotein receptor	LDLR	P01130
LYN proto-oncogene, Src family tyrosine kinase	LYN	P07948

Target name	Gene Name	UniProt ID
lysozyme	LYZ	P61626
macrophage stimulating 1	MST1	ENSG00000173531
MAF bZIP transcription factor F	MAFF	Q9ULX9
major facilitator superfamily domain containing 1	MFSD1	Q9H3U5
major facilitator superfamily domain containing 3	MFSD3	Q96ES6
malic enzyme 1	ME1	P48163
malic enzyme 2	ME2	P23368
Maltase-glucoamylase, intestinal	MGAM	O43451
mannan binding lectin serine peptidase 1	MASP1	P48740
mannan binding lectin serine peptidase 2	MASP2	O00187
maternal embryonic leucine zipper kinase	MELK	Q14680
matrix metallopeptidase 1	MMP1	P03956
matrix metallopeptidase 13	MMP13	P45452
matrix metallopeptidase 2	MMP2	P08253
matrix metallopeptidase 3	MMP3	P08254
matrix metallopeptidase 7	MMP7	P09237
matrix metallopeptidase 8	MMP8	P22894
matrix metallopeptidase 9	MMP9	P14780
Matrix metalloproteinase-9	MMP9	P14780
Matrix metalloproteinase-9	MMP9	P14780
MCL1 apoptosis regulator, BCL2 family member	MCL1	Q07820
megakaryocyte-associated tyrosine kinase	MATK	P42679
melanocyte inducing transcription factor	MITF	O75030
Melatonin receptor type 1A	MTNR1A	P48039
membrane bound transcription factor peptidase, site 1	MBTPS1	Q14703

Target name	Gene Name	UniProt ID
membrane frizzled-related protein	MFRP	Q9BY79
MER proto-oncogene, tyrosine kinase	MERTK	Q12866
MET proto-oncogene, receptor tyrosine kinase	MET	P08581
methionyl aminopeptidase 2	METAP2	P50579
methylsterol monooxygenase 1	MSMO1	Q15800
microsomal glutathione S-transferase 1	MGST1	P10620
microtubule associated protein 1 light chain 3 alpha	MAP1LC3A	Q9H492
microtubule associated protein 2	MAP2	P11137
Microtubule-associated protein 2	MAP2	P11137
mindbomb E3 ubiquitin protein ligase 1	MIB1	Q86YT6
mindbomb E3 ubiquitin protein ligase 2	MIB2	Q96AX9
Mineralocorticoid receptor	NR3C2	P08235
Mitochondrial uncoupling protein 2	UCP2	P55851
Mitochondrial uncoupling protein 3	UCP3	P55916
mitogen-activated protein kinase 1	MAPK1	P28482
mitogen-activated protein kinase 11	MAPK11	Q15759
mitogen-activated protein kinase 12	MAPK12	P53778
mitogen-activated protein kinase 13	MAPK13	O15264
mitogen-activated protein kinase 14	MAPK14	Q16539
mitogen-activated protein kinase 15	MAPK15	Q8TD08
mitogen-activated protein kinase 3	MAPK3	P27361
mitogen-activated protein kinase 4	MAPK4	P31152
mitogen-activated protein kinase 6	MAPK6	Q16659
mitogen-activated protein kinase 7	MAPK7	Q13164
mitogen-activated protein kinase 8	MAPK8	P45983

Target name	Gene Name	UniProt ID
mitogen-activated protein kinase 9	MAPK9	P45984
mitogen-activated protein kinase kinase 1	MAP2K1	Q02750
mitogen-activated protein kinase kinase kinase 10	MAP3K10	Q02779
mitogen-activated protein kinase kinase kinase 11	MAP3K11	Q16584
mitogen-activated protein kinase kinase kinase 20	MAP3K20	Q9NYL2
mitogen-activated protein kinase kinase kinase 21	MAP3K21	Q5TCX8
mitogen-activated protein kinase kinase kinase 7	MAP3K7	O43318
mitogen-activated protein kinase kinase kinase 9	MAP3K9	P80192
mixed lineage kinase domain like pseudokinase	MLKL	Q8NB16
monoamine oxidase A	MAOA	P21397
monoamine oxidase B	MAOB	P27338
MOS proto-oncogene, serine/threonine kinase	MOS	P00540
M-phase inducer phosphatase 1	CDC25A	P30304
M-phase inducer phosphatase 2	CDC25B	P30305
M-phase phosphoprotein 8	MPHOSPH8	Q99549
mRNA of PKA Catalytic Subunit C-alpha	PRKACA	P17612
Muscarinic acetylcholine receptor M1	CHRM1	P11229
Muscarinic acetylcholine receptor M2	CHRM2	P08172
Muscarinic acetylcholine receptor M3	CHRM3	P20309
Muscarinic acetylcholine receptor M4	CHRM4	P08173
muscle associated receptor tyrosine kinase	MUSK	O15146
Mu-type opioid receptor	OPRM1	P35372
Mu-type opioid receptor	OPRM1	P35372
Myc proto-oncogene protein	MYC	P01106
MYC proto-oncogene, bHLH transcription factor	MYC	P01106

Target name	Gene Name	UniProt ID
myeloperoxidase	MPO	P05164
NAD(P)H dehydrogenase [quinone] 1	NQO1	P15559
NAD(P)H quinone dehydrogenase 1	NQO1	P15559
NADH:ubiquinone oxidoreductase core subunit V1	NDUFV1	P49821
NADH:ubiquinone oxidoreductase core subunit V2	NDUFV2	P19404
NADPH dependent diflavin oxidoreductase 1	NDOR1	Q9UHB4
NADPH oxidase 3	NOX3	Q9HBY0
NADPH--cytochrome P450 reductase	POR	P16435
Neuromodulin	GAP43	P17677
Neuronal acetylcholine receptor protein, alpha-7 chain	CHRNA7	P36544
Neuronal acetylcholine receptor subunit alpha-2	CHRNA2	Q15822
Neuronal acetylcholine receptor subunit alpha-7	CHRNA7	P36544
Neutrophil cytosol factor 1	NCF1	P14598
NF-kappa-B inhibitor alpha	NFKBIA	P25963
NFKB inhibitor alpha	NFKBIA	P25963
NFKB inhibitor beta	NFKBIB	Q15653
NFKB inhibitor delta	NFKBID	Q8NI38
NFKB inhibitor epsilon	NFKBIE	O00221
NFKB inhibitor zeta	NFKBIZ	Q9BYH8
NFS1 cysteine desulfurase	NFS1	Q9Y697
Nicotinate-nucleotide--dimethylbenzimidazole phosphoribosyltransferase	cobT	Q05603
NIM1 serine/threonine protein kinase	NIM1K	Q8IY84
nitric oxide synthase 1	NOS1	P29475
nitric oxide synthase 2	NOS2	P35228
nitric oxide synthase 3	NOS3	P29474

Target name	Gene Name	UniProt ID
Nitric oxide synthase, endothelial	NOS3	P29474
Nitric oxide synthase, inducible	NOS2	P35228
Nitric-oxide synthase, endothelial	NOS3	P29474
NME/NM23 nucleoside diphosphate kinase 1	NME1	P15531
nodal growth differentiation factor	NODAL	Q96S42
notch receptor 1	NOTCH1	P46531
notch receptor 2	NOTCH2	Q04721
notch receptor 3	NOTCH3	Q9UM47
notch receptor 4	NOTCH4	Q99466
NOTCH regulated ankyrin repeat protein	NRARP	Q7Z6K4
NPC intracellular cholesterol transporter 1	NPC1	O15118
NPC1-like intracellular cholesterol transporter 1	NPC1L1	Q9UHC9
NTPase KAP family P-loop domain containing 1	NKPD1	Q17RQ9
Nuclear factor erythroid 2-related factor 2	NFE2L2	Q16236
nuclear factor kappa B subunit 1	NFKB1	P19838
nuclear factor kappa B subunit 2	NFKB2	Q00653
nuclear factor of activated T cells 3	NFATC3	Q12968
Nuclear factor of activated T-cells, cytoplasmic 3	NFATC3	Q12968
nuclear factor, erythroid 2 like 2	NFE2L2	Q16236
Nuclear receptor coactivator 1	NCOA1	Q15788
Nuclear receptor coactivator 2	NCOA2	Q15596
Nuclear receptor ROR-alpha	RORA	P35398
Nuclear receptor subfamily 1 group I member 2	NR1I2	O75469
Nuclear receptor subfamily 1 group I member 3	NR1I3	E9PCF2
Nuclear receptor subfamily 1 group I member 3	NR1I3	E9PCF2

Target name	Gene Name	UniProt ID
nuclear receptor subfamily 3 group C member 1	NR3C1	P04150
nuclear respiratory factor 1	NRF1	Q16656
nudix hydrolase 12	NUDT12	Q9BQG2
O-6-methylguanine-DNA methyltransferase	MGMT	P16455
occludin	OCLN	Q16625
oleoyl-ACP hydrolase	OLAH	Q9NV23
opioid receptor delta 1	OPRD1	P41143
opioid receptor mu 1	OPRM1	P35372
ornithine aminotransferase	OAT	P04181
Ornithine decarboxylase	ODC1	P11926
Osteopontin	SPP1	P10451
otoconin 90	OC90	Q02509
ovochymase 1	OVCH1	Q7RTY7
Oxysterols receptor LXR-alpha	NR1H3	Q13133
P2X purinoceptor 3	P2RX3	P56373
p53-induced death domain protein 1	PIDD1	Q9HB75
Pancreas/duodenum homeobox protein 1	PDX1	P52945
paraoxonase 1	PON1	P27169
PAS domain containing serine/threonine kinase	PASK	Q96RG2
PDZ binding kinase	PBK	Q96KB5
Peptide YY	PYY	P10082-2
Peptidyl-glycine alpha-amidating monooxygenase	PAM	P19021
Peroxidase C1A	PRXC1A	P00433
peroxiredoxin 2	PRDX2	P32119
peroxiredoxin 6	PRDX6	P30041

Target name	Gene Name	UniProt ID
peroxisome proliferator activated receptor alpha	PPARA	Q07869
peroxisome proliferator activated receptor delta	PPARD	Q03181
peroxisome proliferator activated receptor gamma	PPARG	P37231
Peroxisome proliferator-activated receptor alpha	PPARA	Q07869
Peroxisome proliferator-activated receptor delta	PPARD	Q03181
Peroxisome proliferator-activated receptor gamma	PPARG	P37231
Peroxisome proliferator-activated receptor gamma	PPARG	P37231
phenylethanolamine N-methyltransferase	PNMT	P11086
phosphatidylcholine transfer protein	PCTP	Q9UKL6
Phosphatidylinositol-3,4,5-trisphosphate 3-phosphatase and dual-specificity protein phosphatase PTEN	PTEN	P60484
phosphatidylinositol-4,5-bisphosphate 3-kinase catalytic subunit alpha	PIK3CA	P42336
phosphatidylinositol-4,5-bisphosphate 3-kinase catalytic subunit beta	PIK3CB	P42338
phosphatidylinositol-4,5-bisphosphate 3-kinase catalytic subunit delta	PIK3CD	O00329
phosphatidylinositol-4,5-bisphosphate 3-kinase catalytic subunit gamma	PIK3CG	P48736
Phosphatidylinositol-4,5-bisphosphate 3-kinase catalytic subunit, gamma isoform	PIK3CG	P48736
phosphodiesterase 3A	PDE3A	Q14432
phosphoethanolamine/phosphocholine phosphatase 1	PHOSPHO1	Q8TCT1
phosphoglucomutase 1	PGM1	P36871
phosphoglycolate phosphatase	PGP	A6NDG6
phosphoinositide-3-kinase regulatory subunit 1	PIK3R1	P27986
phospholipase A2 group IB	PLA2G1B	P04054
phospholipase A2 group IIA	PLA2G2A	P14555
phospholipase A2 group IIC	PLA2G2C	Q5R387
phospholipase A2 group IID	PLA2G2D	Q9UNK4
phospholipase A2 group IIE	PLA2G2E	Q9NZK7

Target name	Gene Name	UniProt ID
phospholipase A2 group IIF	PLA2G2F	Q9BZM2
phospholipase A2 group V	PLA2G5	P39877
phospholipase A2 group VI	PLA2G6	O60733
phospholipase A2 group X	PLA2G10	O15496
phospholipase B1	PLB1	Q6P1J6
Pim-1 proto-oncogene, serine/threonine kinase	PIM1	P11309
Pim-2 proto-oncogene, serine/threonine kinase	PIM2	Q9P1W9
Pim-3 proto-oncogene, serine/threonine kinase	PIM3	Q86V86
pipecolic acid and sarcosine oxidase	PIPOX	Q9P0Z9
plasminogen	PLG	P00747
Plasminogen activator inhibitor 1	SERPINE1	P05121
plasminogen activator, tissue type	PLAT	P00750
plasminogen activator, urokinase	PLAU	P00749
plasminogen activator, urokinase receptor	PLAUR	Q03405
platelet activating factor acetylhydrolase 1b catalytic subunit 3	PAFAH1B3	Q15102
platelet derived growth factor receptor alpha	PDGFRA	P16234
platelet derived growth factor receptor beta	PDGFRB	P09619
platelet derived growth factor receptor like	PDGFRL	Q15198
Poly [ADP-ribose] polymerase 1	PARP1	P09874
poly(ADP-ribose) polymerase 1	PARP1	P09874
Polyunsaturated fatty acid 5-lipoxygenase	ALOX5	P09917
Polyunsaturated fatty acid lipoxygenase ALOX12	ALOX12	P18054
Polyunsaturated fatty acid lipoxygenase ALOX15	ALOX15	P16050
Potassium voltage-gated channel subfamily A member 3	KCNA3	P22001
Potassium voltage-gated channel subfamily H member 2	KCNH2	Q12809

Target name	Gene Name	UniProt ID
potassium voltage-gated channel subfamily Q member 1	KCNQ1	P51787
POTE ankyrin domain family member B	POTEB	A0A0A6YYL3
POTE ankyrin domain family member B2	POTEB2	H3BUK9
POTE ankyrin domain family member C	POTEC	B2RU33
POTE ankyrin domain family member D	POTED	Q86YR6
POTE ankyrin domain family member E	POTEE	Q6S8J3
POTE ankyrin domain family member H	POTEH	Q6S545
POTE ankyrin domain family member I	POTEI	P0CG38
POTE ankyrin domain family member J	POTEJ	P0CG39
Probable E3 ubiquitin-protein ligase HERC5	HERC5	Q9UII4
Procollagen C-endopeptidase enhancer 1	PCOLCE	Q15113
Pro-epidermal growth factor	EGF	P01133
Progesterone receptor	PGR	P06401
proopiomelanocortin	POMC	P01189
proprotein convertase subtilisin/kexin type 1	PCSK1	P29120
proprotein convertase subtilisin/kexin type 2	PCSK2	P16519
proprotein convertase subtilisin/kexin type 4	PCSK4	Q6UW60
proprotein convertase subtilisin/kexin type 5	PCSK5	Q92824
proprotein convertase subtilisin/kexin type 7	PCSK7	Q16549
proprotein convertase subtilisin/kexin type 9	PCSK9	Q8NBP7
prostaglandin E receptor 4	PTGER4	P35408
Prostaglandin E2 receptor EP3 subtype	PTGER3	P43115
Prostaglandin G/H synthase 1	PTGS1	P23219
Prostaglandin G/H synthase 2	PTGS2	P35354
Prostaglandin G/H synthase 2	PTGS2	P35354

Target name	Gene Name	UniProt ID
prostaglandin I2 synthase	PTGIS	Q16647
prostaglandin-endoperoxide synthase 1	PTGS1	P23219
prostaglandin-endoperoxide synthase 2	PTGS2	P35354
Prostatic acid phosphatase	ACP3	P15309
proteasome 26S subunit, non-ATPase 10	PSMD10	O75832
protein C, inactivator of coagulation factors Va and VIIIa	PROC	P04070
Protein CBFA2T1	RUNX1T1	Q06455
protein kinase C alpha	PRKCA	P17252
Protein kinase C alpha type	PRKCA	P17252
protein kinase C beta	PRKCB	P05771
Protein kinase C beta type	PRKCB	P05771
protein kinase C delta	PRKCD	Q05655
protein kinase C epsilon	PRKCE	Q02156
Protein kinase C epsilon type	PRKCE	Q02156
protein kinase C eta	PRKCH	P24723
protein kinase C gamma	PRKCG	P05129
protein kinase C theta	PRKCQ	Q04759
protein kinase domain containing, cytoplasmic	PKDCC	Q504Y2
protein kinase N1	PKN1	Q16512
protein kinase N2	PKN2	Q16513
protein kinase N3	PKN3	Q6P5Z2
protein kinase, DNA-activated, catalytic subunit	PRKDC	P78527
protein phosphatase 1 regulatory subunit 12A	PPP1R12A	O14974
protein phosphatase 1 regulatory subunit 12C	PPP1R12C	Q9BZL4
protein phosphatase 1 regulatory subunit 16A	PPP1R16A	Q96I34

Target name	Gene Name	UniProt ID
protein phosphatase 1 regulatory subunit 16B	PPP1R16B	Q96T49
protein phosphatase 1 regulatory subunit 27	PPP1R27	Q86WC6
protein tyrosine kinase 2	PTK2	Q05397
protein tyrosine kinase 6	PTK6	Q13882
protein tyrosine kinase 7 (inactive)	PTK7	Q13308
protein tyrosine phosphatase non-receptor type 1	PTPN1	P18031
Protein tyrosine phosphatase receptor type C-associated protein	PTPRCAP	Q14761
protein Z, vitamin K dependent plasma glycoprotein	PROZ	P22891
proteinase 3	PRTN3	P24158
Proto-oncogene c-Fos	FOS	P01100
purine nucleoside phosphorylase	PNP	P00491
Puromycin-sensitive aminopeptidase	NPEPPSL1	A6NEC2
pyridoxal phosphatase	PDXP	Q96GD0
pyrroline-5-carboxylate reductase 1	PYCR1	P32322
pyrroline-5-carboxylate reductase 2	PYCR2	Q96C36
pyruvate carboxylase	PC	P11498
pyruvate dehydrogenase E1 subunit beta	PDHB	P11177
pyruvate kinase L/R	PKLR	P30613
pyruvate kinase M1/2	PKM	P14618
Rac family small GTPase 1	RAC1	P63000
RAC-alpha serine/threonine-protein kinase	AKT1	P31749
RAF proto-oncogene serine/threonine-protein kinase	RAF1	P04049
Ras association domain-containing protein 1	RASSF1	Q9NS23
Ras GTPase-activating protein 1	RASA1	P20936
ras homolog family member A	RHOA	P61586

Target name	Gene Name	UniProt ID
ras homolog family member B	RHOB	P62745
receptor interacting serine/threonine kinase 1	RIPK1	Q13546
receptor interacting serine/threonine kinase 2	RIPK2	O43353
receptor interacting serine/threonine kinase 3	RIPK3	Q9Y572
receptor interacting serine/threonine kinase 4	RIPK4	P57078
receptor tyrosine kinase like orphan receptor 1	ROR1	Q01973
Receptor tyrosine-protein kinase erbB-2	ERBB2	P04626
Receptor tyrosine-protein kinase erbB-3	ERBB3	P21860
regulatory factor X associated ankyrin containing protein	RFXANK	O14593
RELA proto-oncogene, NF-kB subunit	RELA	Q04206
ret proto-oncogene	RET	P07949
Retinoblastoma-associated protein	RB1	P06400
retinoic acid induced 14	RAI14	Q9P0K7
Retinoic acid receptor RXR-alpha	RXRA	A0A3B3IS44
retinoid X receptor alpha	RXRA	A0A3B3IS44
retinoid X receptor beta	RXRB	P28702
Retinol-binding protein 2	RBP2	P50120
Retinol-binding protein 4	RBP4	P02753
rhodopsin	RHO	P08100
ribokinase	RBKS	Q9H477
ribonuclease A family member 1, pancreatic	RNASE1	P07998
ribonuclease L	RNASEL	Q05823
ribonucleotide reductase catalytic subunit M1	RRM1	P23921
ribosomal protein S6 kinase A3	RPS6KA3	P51812
Ribosyldihydronicotinamide dehydrogenase [quinone]	NQO2	P16083

Target name	Gene Name	UniProt ID
RNA 3'-terminal phosphate cyclase	RTCA	O00442
R-spondin 1	RSPO1	Q2MKA7
R-spondin 3	RSPO3	Q9BXY4
R-spondin 4	RSPO4	Q2I0M5
Runt-related transcription factor 2	RUNX2	Q13950
S100 calcium binding protein A4	S100A4	P26447
secreted frizzled related protein 1	SFRP1	Q8N474
secreted frizzled related protein 2	SFRP2	Q96HF1
secreted frizzled related protein 4	SFRP4	Q6FHJ7
secreted frizzled related protein 5	SFRP5	Q5T4F7
Sentrin-specific protease 6	SENP6	Q9GZR1
Sentrin-specific protease 8	SENP8	Q96LD8
serine dehydratase	SDS	P20132
serine hydroxymethyltransferase 1	SHMT1	P34896
serine hydroxymethyltransferase 2	SHMT2	P34897
serine protease 1	PRSS1	P07477
serine protease 12	PRSS12	P56730
serine protease 21	PRSS21	Q9Y6M0
serine protease 22	PRSS22	Q9GZN4
serine protease 27	PRSS27	Q9BQR3
serine protease 3	PRSS3	P35030
serine protease 33	PRSS33	Q8NF86
serine protease 36	PRSS36	Q5K4E3
serine protease 37	PRSS37	A4D1T9
serine protease 38	PRSS38	A1L453

Target name	Gene Name	UniProt ID
serine protease 40A (pseudogene)	PRSS40A	#N/A
serine protease 42, pseudogene	PRSS42P	#N/A
serine protease 45, pseudogene	PRSS45P	Q7RTY3
serine protease 46, pseudogene	PRSS46P	#N/A
serine protease 48	PRSS48	Q7RTY5
serine protease 50	PRSS50	ENSG00000283706
serine protease 53	PRSS53	Q2L4Q9
serine protease 54	PRSS54	Q6PEW0
serine protease 55	PRSS55	Q6UWB4
serine protease 57	PRSS57	Q6UWY2
serine protease 58	PRSS58	Q8IYP2
serine protease 8	PRSS8	Q16651
serine/threonine kinase 11	STK11	Q15831
serine/threonine kinase 40	STK40	Q8N2I9
serine/threonine/tyrosine kinase 1	STYK1	Q6J9G0
Serine/threonine-protein kinase Chk2	CHEK2	O96017
serpin family E member 1	SERPINE1	P05121
Serum paraoxonase/arylesterase 1	PON1	P27169
Sex hormone-binding globulin	SHBG	P04278
SH3 and multiple ankyrin repeat domains 1	SHANK1	Q9Y566
SH3 and multiple ankyrin repeat domains 2	SHANK2	Q9UPX8
Short transient receptor potential channel 6	TRPC6	Q9Y210
sigma non-opioid intracellular receptor 1	SIGMAR1	Q99720
Sigma non-opioid intracellular receptor 1	SIGMAR1	Q99720
signal transducer and activator of transcription 1	STAT1	P42224

Target name	Gene Name	UniProt ID
Signal transducer and activator of transcription 1-alpha/beta	STAT1	P42224
Signal transducer and activator of transcription 1-alpha/beta	STAT1	P42224
signal transducer and activator of transcription 3	STAT3	P40763
sirtuin 1	SIRT1	Q96EB6
small ubiquitin like modifier 4	SUMO4	Q6EEV6
SMC5-SMC6 complex localization factor 1	SLF1	Q9BQI6
smoothened, frizzled class receptor	SMO	Q99835
Sodium channel protein type 5 subunit alpha	SCN5A	Q14524
sodium voltage-gated channel alpha subunit 5	SCN5A	Q14524
Sodium-dependent dopamine transporter	SLC6A3	Q01959
Sodium-dependent dopamine transporter	SLC6A3	Q01959
Sodium-dependent noradrenaline transporter	SLC6A2	P23975
Sodium-dependent noradrenaline transporter	SLC6A2	P23975
Sodium-dependent serotonin transporter	SLC6A4	P31645
Sodium-dependent serotonin transporter	SLC6A4	P31645
solute carrier family 12 member 2	SLC12A2	P55011
solute carrier family 16 member 1	SLC16A1	P53985
solute carrier family 16 member 10	SLC16A10	Q8TF71
solute carrier family 16 member 11	SLC16A11	Q8NCK7
solute carrier family 16 member 12	SLC16A12	Q6ZSM3
solute carrier family 16 member 5	SLC16A5	O15375
solute carrier family 16 member 6	SLC16A6	O15403
solute carrier family 16 member 8	SLC16A8	O95907
solute carrier family 16 member 9	SLC16A9	Q7RTY1
solute carrier family 17 member 3	SLC17A3	O00476

Target name	Gene Name	UniProt ID
solute carrier family 2 member 1	SLC2A1	P11166
solute carrier family 2 member 11	SLC2A11	Q9BYW1
solute carrier family 2 member 14	SLC2A14	Q8TDB8
solute carrier family 2 member 2	SLC2A2	P11168
solute carrier family 2 member 3	SLC2A3	P11169
solute carrier family 2 member 4	SLC2A4	P14672
solute carrier family 2 member 5	SLC2A5	P22732
solute carrier family 2 member 7	SLC2A7	Q6PXP3
solute carrier family 2 member 9	SLC2A9	Q9NRM0
Solute carrier family 2, facilitated glucose transporter member 2	SLC2A2	P11168
Solute carrier family 2, facilitated glucose transporter member 4	SLC2A4	P14672
solute carrier family 22 member 1	SLC22A1	O15245
Solute carrier family 22 member 1	SLC22A1	O15245
solute carrier family 22 member 10	SLC22A10	Q63ZE4
solute carrier family 22 member 11	SLC22A11	Q9NSA0
solute carrier family 22 member 12	SLC22A12	Q96S37
solute carrier family 22 member 2	SLC22A2	O15244
solute carrier family 22 member 24	SLC22A24	Q8N4F4
solute carrier family 22 member 25	SLC22A25	Q6T423
Solute carrier family 22 member 3	SLC22A3	O75751
solute carrier family 22 member 6	SLC22A6	Q4U2R8
solute carrier family 22 member 7	SLC22A7	Q9Y694
solute carrier family 22 member 8	SLC22A8	Q8TCC7
solute carrier family 22 member 9	SLC22A9	Q8IVM8
solute carrier family 25 member 10	SLC25A10	Q9UBX3

Target name	Gene Name	UniProt ID
solute carrier family 33 member 1	SLC33A1	O00400
solute carrier family 36 member 1	SLC36A1	Q7Z2H8
solute carrier family 39 member 5	SLC39A5	Q6ZMH5
solute carrier family 6 member 2	SLC6A2	P23975
solute carrier family 6 member 3	SLC6A3	Q01959
solute carrier family 6 member 4	SLC6A4	P31645
sosondowah ankyrin repeat domain family member C	SOWAHC	Q53LP3
sphingolipid transporter 1 (putative)	SPNS1	Q9H2V7
sphingolipid transporter 2	SPNS2	Q8IVW8
sphingolipid transporter 3 (putative)	SPNS3	Q6ZMD2
Sphingosine 1-phosphate receptor 1	S1PR1	P21453
Spi-1 proto-oncogene	SPI1	P17947
spleen associated tyrosine kinase	SYK	P43405
SRC proto-oncogene, non-receptor tyrosine kinase	SRC	P12931
src-related kinase lacking C-terminal regulatory tyrosine and N-terminal myristylation sites	SRMS	Q9H3Y6
ST14 transmembrane serine protease matriptase	ST14	Q9Y5Y6
Steroid 17-alpha-hydroxylase/17,20 lyase	CYP17A1	P05093
Steroidogenic factor 1	NR5A1	Q13285
sterol carrier protein 2	SCP2	P22307
Sterol O-acyltransferase 1	SOAT1	P35610
sterol regulatory element binding transcription factor 1	SREBF1	P36956
Sterol regulatory element-binding protein 2	SREBF2	Q12772
Stromelysin-1	MMP3	P08254
succinate dehydrogenase complex flavoprotein subunit A	SDHA	P31040
sucrase-isomaltase	SI	P14410

Target name	Gene Name	UniProt ID
sulfotransferase family 1A member 1	SULT1A1	P50225
Superoxide dismutase [Cu-Zn]	SOD1	P00441
suprabasin	SBSN	Q6UWP8
synaptic vesicle glycoprotein 2B	SV2B	Q7L1I2
syndecan 3	SDC3	O75056
synuclein alpha	SNCA	P37840
synuclein alpha interacting protein	SNCAIP	Q9Y6H5
tachykinin precursor 1	TAC1	P20366
tachykinin receptor 1	TACR1	P25103
tankyrase	TNKS	O95271
tankyrase 2	TNKS2	Q9H2K2
TEK receptor tyrosine kinase	TEK	Q02763
Telomerase protein component 1	TEP1	Q99973
telomerase reverse transcriptase	TERT	O14746
testis associated actin remodelling kinase 1	TESK1	Q15569
testis associated actin remodelling kinase 2	TESK2	Q96S53
testis specific serine kinase 1B	TSSK1B	Q9BXA7
testis specific serine kinase 2	TSSK2	Q96PF2
testis specific serine kinase 3	TSSK3	Q96PN8
testis specific serine kinase 4	TSSK4	Q6SA08
tetratricopeptide repeat, ankyrin repeat and coiled-coil containing 1	TANC1	Q9C0D5
tetratricopeptide repeat, ankyrin repeat and coiled-coil containing 2	TANC2	Q9HCD6
Thrombin	F2	P00734
Thrombomodulin	THBD	P07204
thromboxane A synthase 1	TBXAS1	P24557

Target name	Gene Name	UniProt ID
TIMP metallopeptidase inhibitor 1	TIMP1	P01033
TIMP metallopeptidase inhibitor 3	TIMP3	P35625
Tissue factor	F3	P13726
Tissue-type plasminogen activator	PLAT	P00750
TNF receptor associated factor 1	TRAF1	Q13077
TNF receptor associated factor 3	TRAF3	Q13114
TNF receptor associated factor 4	TRAF4	Q9BUZ4
TNF receptor superfamily member 10a	TNFRSF10A	O00220
TNF receptor superfamily member 10b	TNFRSF10B	O14763
TNF receptor superfamily member 1A	TNFRSF1A	P19438
TNF receptor superfamily member 25	TNFRSF25	Q93038
TNF superfamily member 10	TNFSF10	P50591
TNF superfamily member 13b	TNFSF13B	Q9Y275
toll like receptor 4	TLR4	O00206
tonsoku like, DNA repair protein	TONSL	Q96HA7
trafficking protein particle complex 4	TRAPPC4	Q9Y296
Transcription factor AP-1	JUN	P05412
Transcription factor E2F1	E2F1	Q01094
Transcription factor E2F2	E2F2	Q14209
Transcription factor p65	RELA	Q04206
transforming growth factor alpha	TGFA	P01135
transforming growth factor beta 1	TGFB1	P01137
Transforming growth factor beta-1	TGFB1	P01137
transient erythroblastopenia of childhood	TEC	P42680
transient receptor potential cation channel subfamily A member 1	TRPA1	O75762

Target name	Gene Name	UniProt ID
transient receptor potential cation channel subfamily M member 8	TRPM8	Q7Z2W7
transient receptor potential cation channel subfamily V member 1	TRPV1	Q8NER1
Transient receptor potential cation channel subfamily V member 1	TRPV1	Q8NER1
transient receptor potential cation channel subfamily V member 2	TRPV2	Q9Y5S1
transient receptor potential cation channel subfamily V member 3	TRPV3	Q8NET8
transient receptor potential cation channel subfamily V member 4	TRPV4	Q9HBA0
transient receptor potential cation channel subfamily V member 5	TRPV5	Q9NQA5
transient receptor potential cation channel subfamily V member 6	TRPV6	ENSG00000276971
Translocator protein	TSPO	P30536
transmembrane 7 superfamily member 2	TM7SF2	O76062
transmembrane serine protease 11A	TMPRSS11A	Q6ZMR5
transmembrane serine protease 11B	TMPRSS11B	Q86T26
transmembrane serine protease 11D	TMPRSS11D	O60235
transmembrane serine protease 11E	TMPRSS11E	Q9UL52
transmembrane serine protease 11F	TMPRSS11F	Q6ZWK6
transmembrane serine protease 12	TMPRSS12	Q86WS5
transmembrane serine protease 13	TMPRSS13	Q9BYE2
transmembrane serine protease 15	TMPRSS15	P98073
transmembrane serine protease 2	TMPRSS2	O15393
transmembrane serine protease 3	TMPRSS3	P57727
transmembrane serine protease 4	TMPRSS4	Q9NRS4
transmembrane serine protease 5	TMPRSS5	Q9H3S3
transmembrane serine protease 6	TMPRSS6	Q8IU80
transmembrane serine protease 7	TMPRSS7	Q7RTY8
transmembrane serine protease 9	TMPRSS9	Q7Z410

Target name	Gene Name	UniProt ID
tribbles pseudokinase 1	TRIB1	Q96RU8
tribbles pseudokinase 2	TRIB2	Q92519
tribbles pseudokinase 3	TRIB3	Q96RU7
triokinase and FMN cyclase	TKFC	Q3LXA3
triosephosphate isomerase 1	TPI1	P60174
tripeptidyl peptidase 2	TPP2	P29144
tRNA-yW synthesizing protein 1 homolog	TYW1	Q9NV66
Trypsin-1	PRSS1	P07477
Trypsin-3	PRSS3	P35030
tryptase alpha/beta 1	TPSAB1	Q15661
tryptase delta 1	TPSD1	Q9BZJ3
tryptase gamma 1	TPSG1	Q9NRR2
Tubulin beta-1 chain	TUBB1	Q9H4B7
tumor necrosis factor	TNF	P01375
tumor protein p53	TP53	P04637
TXK tyrosine kinase	TXK	P42681
Type I iodothyronine deiodinase	DIO1	P49895
TYRO3 protein tyrosine kinase	TYRO3	Q06418
tyrosinase	TYR	P14679
tyrosine kinase 2	TYK2	P29597
tyrosine kinase with immunoglobulin like and EGF like domains 1	TIE1	P35590
Tyrosine-protein phosphatase non-receptor type 1	PTPN1	P18031
Tyrosyl-DNA phosphodiesterase 2	TDP2	O95551
UDP glucuronosyltransferase family 2 member B17	UGT2B17	O75795
UDP-galactose-4-epimerase	GALE	Q14376

Target name	Gene Name	UniProt ID
uncoupling protein 1	UCP1	P25874
uncoupling protein 2	UCP2	P55851
Urokinase-type plasminogen activator	PLAU	P00749
uroporphyrinogen decarboxylase	UROD	P06132
uveal autoantigen with coiled-coil domains and ankyrin repeats	UACA	Q9BZF9
vacuole membrane protein 1	VMP1	Q96GC9
vascular cell adhesion molecule 1	VCAM1	P19320
Vascular cell adhesion protein 1	VCAM1	P19320
vascular endothelial growth factor A	VEGFA	P15692
Wnt family member 5A	WNT5A	P41221
xanthine dehydrogenase	XDH	P47989
Xanthine dehydrogenase/oxidase	XDH	P47989
X-box-binding protein 1	XBP1	P17861
YES proto-oncogene 1, Src family tyrosine kinase	YES1	P07947
zeta chain of T cell receptor associated protein kinase 70	ZAP70	P43403
zinc finger DHHC-type palmitoyltransferase 13	ZDHHC13	Q8IUH4
zinc finger DHHC-type palmitoyltransferase 17	ZDHHC17	Q8IUH5
