# Supplementary material for: Therapeutic Effects of Zanthoxyli Pericarpium on Intestinal Inflammation and Network Pharmacological Mechanism Analysis in a Dextran Sodium Sulfate-Induced Colitis Mouse Model
Source: Nutrients. 2024 Oct 17;16(20):3521. doi: 10.3390/nu16203521 (PMC11510417; doi:10.3390/nu16203521)
Supplement: Supplementary file 1 [file nutrients-16-03521-s001.zip › Table S6 Disease related 100 genes.pdf]

Table S6. 100 Disease–related genes

Gene name	Protein name
IRGM	LPS-stimulated RAW 264.7 macrophage protein 47 homolog
CLDN5	Transmembrane protein deleted in VCFS
CLDN4	Williams-Beuren syndrome chromosomal region 8 protein
FFAR3	G-protein coupled receptor 41
SMAD7	Mothers against decapentaplegic homolog 7
CLDN3	Clostridium perfringens enterotoxin receptor 2
FFAR2	G-protein coupled receptor 43
MEFV	Mediterranean fever
RIPK2	CARD-containing interleukin-1 beta-converting enzyme-associated kinase
SLC37A4	Solute carrier family 37 (glucose-6-phosphate transporter), member 4
TLR5	Toll/interleukin-1 receptor-like protein 3
TLR2	Toll/interleukin-1 receptor-like protein 4
JAK2	Tyrosine-protein kinase JAK2
LGR5	Leucine-rich repeat containing G protein-coupled receptor 5
TNFSF15	Tumor necrosis factor (ligand) superfamily, member 15
IL33	Nuclear factor from high endothelial venules
HP	Haptoglobin
TNF	Tumor necrosis factor ligand superfamily member 2
IFNG	Immune interferon
IL1B	Interleukin 1, beta
CD4	T-cell surface antigen T4/Leu-3
CD8A	T-lymphocyte differentiation antigen T8/Leu-2
HLA-DQA2	HLA class II histocompatibility antigen, DQ(6) alpha chain
CRP	C-reactive protein, pentraxin-related
LRG1	Leucine rich alpha-2-glycoprotein 1
CXCL10	10 kDa interferon gamma-induced protein
CSF2	Colony stimulating factor 2 (granulocyte-macrophage)
IL4	Lymphocyte stimulatory factor 1
IL5	Eosinophil differentiation factor
MPO	Myeloperoxidase
IL6	B-cell stimulatory factor 2
ICAM1	Intercellular adhesion molecule 1
CXCL1	Chemokine (C-X-C motif) ligand 1 (melanoma growth stimulating activity, alpha)
HMOX1	Heme oxygenase (decycling) 1
CDH1	Cadherin 1, type 1, E-cadherin (epithelial)
CCL2	Monocyte chemotactic and activating factor
IDO1	Indoleamine-pyrrole 2,3-dioxygenase
IL9	T-cell growth factor P40
PTPN2	Protein tyrosine phosphatase, non-receptor type 2
AOC1	Amiloride-sensitive amine oxidase [copper-containing]
CXCL2	Macrophage inflammatory protein 2-alpha
ATP4A	ATPase, H+/K+ exchanging, alpha polypeptide

Gene name	Protein name
ITGAX	Integrin, alpha X (complement component 3 receptor 4 subunit)
IL10	Cytokine synthesis inhibitory factor
JAK1	Tyrosine-protein kinase JAK1
CXCR2	High affinity interleukin-8 receptor B
NFKBIA	Nuclear factor of kappa light polypeptide gene enhancer in B-cells inhibitor, alpha
CASP1	Caspase 1, apoptosis-related cysteine peptidase
TYK2	Non-receptor tyrosine-protein kinase TYK2
AKT1	V-akt murine thymoma viral oncogene homolog 1
CTNNB1	Catenin (cadherin-associated protein), beta 1, 88kDa
IL13	Interleukin 13
PTGS2	Prostaglandin-endoperoxide synthase 2 (prostaglandin G/H synthase and cyclooxygenase)
STAT3	Signal transducer and activator of transcription 3 (acute-phase response factor)
STAT1	Signal transducer and activator of transcription 1-alpha/beta
CASP3	Caspase 3, apoptosis-related cysteine peptidase
LRBA	LPS-responsive vesicle trafficking, beach and anchor containing
ATP12A	ATPase, H+/K+ transporting, nongastric, alpha polypeptide
CLDN8	Claudin 8
CLDN2	Claudin 2
GSDMD	Gasdermin domain-containing protein 1
NLRP6	NACHT, LRR and PYD domains-containing protein 6
IL2	T-cell growth factor
CCL20	Liver and activation-regulated chemokine
LCN2	25 kDa alpha-2-microglobulin-related subunit of MMP-9
TJP1	Tight junction protein ZO-1
HLA-DQA1	Major histocompatibility complex, class II, DQ alpha 1
MADCAM1	Mucosal vascular addressin cell adhesion molecule 1
IL10RA	Interleukin-10 receptor subunit alpha
IL18	Interferon gamma-inducing factor
PPARG	Peroxisome proliferator-activated receptor gamma
IL17A	Cytotoxic T-lymphocyte-associated antigen 8
OCLN	Occludin
TMEM201	Spindle-associated membrane protein 1
TPMT	Thiopurine S-methyltransferase
TLR4	Toll-like receptor 4
ALB	Serum albumin
IL23R	Interleukin 23 receptor
GPR65	T-cell death-associated gene 8 protein
GPBAR1	G protein-coupled bile acid receptor 1
SLCO2A1	Solute carrier organic anion transporter family, member 2A1
NLRP3	Cold-induced autoinflammatory syndrome 1 protein
CTLA4	Cytotoxic T-lymphocyte-associated antigen 4
IL17F	Interleukin 17F
NR1H4	Nuclear receptor subfamily 1, group H, member 4
MYD88	Myeloid differentiation primary response protein MyD88

**Gene name**	**Protein name**
CLEC7A	Dendritic cell-associated C-type lectin 1
FOXP3	Forkhead box protein P3
IL22	IL-10-related T-cell-derived-inducible factor
CARD9	Caspase recruitment domain-containing protein 9
ATG16L1	Autophagy related 16-like 1 (S. cerevisiae)
IL1RL2	Interleukin-1 receptor-related protein 2
NOD2	Nucleotide-binding oligomerization domain-containing protein 2
IL26	Interleukin 26
NUDT15	Nucleoside diphosphate-linked to another moiety X hydrolase 15
CXCL8	Monocyte-derived neutrophil chemotactic factor
TJP2	Tight junction protein ZO-2
MYLK	Myosin light chain kinase, smooth muscle
TTC7A	Tetratricopeptide repeat protein 7A
F11R	Junctional adhesion molecule 1
